# Towards data-driven predictive maintenance for vacuum ion pumps at European XFEL

**DOI:** 10.1107/S1600577526009197

**Published:** 2026-10-08

**Authors:** Amna Majid, Michaela Petrich, Martin Dommach, Steve Aplin, Walid Maalej, Luca Gelisio, Danilo Ferreira de Lima

**Affiliations:** a European X-ray Free Electron Laser Facility, Schenefeld, Germany; b Hasso Plattner Institute University of Potsdam Germany; ESRF – The European Synchrotron, France

**Keywords:** large-scale scientific facilities, predictive maintenance, vacuum pumps, early fault detection, machine learning, sputter ion pumps, noble gas instability

## Abstract

Large-scale scientific facilities, such as the European XFEL, are highly complex, comprising multiple subsystems that must operate in coordination to produce high-quality scientific output. Any fault within such subsystems can cause unexpected interruptions, with a significant impact on the scientific output. Therefore, it is fundamental to detect abnormal behavior in components well in advance, allowing for timely interventions and efficient maintenance planning. Vacuum ion pumps are an integral part of these facilities, and their smooth operation is essential to maintain overall performance. However, monitoring a large number of pumps is challenging and requires significant human efforts. In this paper, we propose the application of machine-learning techniques to develop an early fault detection methodology for vacuum ion pumps. We conducted several studies to investigate the utilization of the Support Vector Machine and Convolutional Neural Networks to classify pressure data obtained from multiple vacuum ion pumps installed at the European XFEL. In addition, we tackle a common issue while collecting the training data from reliable subsystems, that is, the rarity of fault-related examples. This can hinder the model’s ability to generalize effectively, potentially leading to poor performance when deployed on unseen data. To address these challenges, we investigate two methods, cost-sensitive learning and the Synthetic Minority Over-sampling Technique (SMOTE), in order to improve classification performance on imbalanced datasets. Our developed system shows promising results and significantly automates the fault identification process with an F1-score of 80%, thus reducing manual efforts.

## Abstract 2

This paper presents a machine-learning-based approach for early fault detection in vacuum ion pumps at the European XFEL by analyzing pressure data to detect fault precursors. The developed method enables predictive maintenance, reducing unplanned downtime and improving operational reliability.

## 1. Introduction

Large scientific facilities are complex and include many interconnected devices and components that work in precise coordination (Rauch *et al.*, 2020 ▸; Noack *et al.*, 2021 ▸). These components may gradually deviate from their intended behavior due to factors such as deployment conditions, aging, or external environmental influences. A fault occurring in a particular section can propagate to others, risking the stability of the entire system (Apollonio, 2015 ▸). The growing complexity of such systems underscores the need to develop effective fault prediction strategies to optimize maintenance processes and ensure long-term reliability (Pagano *et al.*, 2012 ▸; Carvalho *et al.*, 2019 ▸). The emergence of machine-learning (ML) techniques, coupled with increased computational resources and access to vast amounts of equipment data, suggests a promising avenue to explore data-driven fault prediction strategies for enhanced operational efficiency (Carvalho *et al.*, 2019 ▸; Nassif *et al.*, 2021 ▸).

The existing literature has explored the application of ML techniques across various components and subsystems of large-scale scientific facilities (Radaideh *et al.*, 2023 ▸; Reščič *et al.*, 2022 ▸; Guler *et al.*, 2022 ▸; Branlard *et al.*, 2022 ▸; Sulc *et al.*, 2023 ▸; Nawaz *et al.*, 2021 ▸; Rahman *et al.*, 2024 ▸); however, there is relatively limited research focused specifically on fault prediction for the vacuum system, which creates and maintains the gas-free environment that is necessary to operate large-scale photon sources. The vacuum system in such facilities is particularly unique because they operate under ultra-high-vacuum conditions, which are significantly different from standard vacuum applications, presenting therefore distinct challenges.

Vacuum pumps, specifically sputter ion pumps (SIPs), are closed pumps and have a limitation in the amount of noble gases, like argon, xenon or krypton, that they can pump before experiencing a fault. Piekarski *et al.* (2020 ▸) proposed the utilization of convolutional neural networks for analysis of pressure data from multiple vacuum pump controllers to detect anomalous events at the Solaris National Synchrotron Radiation Center. However, this work addresses the issue as a generic anomaly detection problem to detect beam instabilities and does not focus on identifying faults specific to SIPs themselves.

In this paper, we present the development of an early fault detection system for SIPs installed at the European XFEL. For this task, we analyze the pressure data obtained from multiple pumps and investigate the application of two ML approaches to classify these data: support vector machine (SVM) and convolutional neural networks (CNNs). SVM belongs to the family of classical ML algorithms and relies on manual feature engineering, whereas CNNs are part of the deep-learning paradigm and automatically learn complex features from raw data.

A major challenge in the training data from SIPs at the European XFEL lies in their highly imbalanced nature. Instances corresponding to fault precursors are extremely rare, representing only about 0.7% of the total data collected for this study, with the vast majority corresponding to normal operation. This imbalance complicates model training, as it tends to be biased towards the majority class, resulting in poor detection of minority-class fault precursors and potential underfitting (He & Garcia, 2009 ▸). To address this issue, we conducted an in-depth study of techniques for handling imbalanced datasets. Specifically, we examined two strategies: cost-sensitive learning and the Synthetic Minority Over-Sampling Technique (SMOTE).

The paper is structured as follows. In Section 2 we provide a brief overview of the vacuum system of the European XFEL facility, and the specific fault type in SIPs investigated in this study. Section 3 describes the data collection and preparation steps. In Section 4 we provide details about the ML models for supervised classification along with the techniques used to handle imbalanced data. Section 5 discusses experiments and their results. Finally, a summary of the paper is presented in Section 6.

## 2. Vacuum system of European XFEL’s photon beamlines

The European XFEL is a large-scale scientific facility, located in Germany, that produces ultrashort and extremely intense X-ray pulses to study matter at the atomic and molecular levels (Decking *et al.*, 2020 ▸; Tschentscher, 2023 ▸). The vacuum system is essential for any large-scale photon source facility and it is responsible for achieving and maintaining an ultra-high vacuum level (of the order of 1 × 10^−9^ mbar) within the photon beamline.

The ultra-high level of vacuum is crucial to ensure the smooth operation and quality of produced output. Any deterioration in vacuum levels can cause a pressure breach that can disrupt the operation, resulting in loss of beam uptime. Therefore, proper functioning of vacuum pumps is extremely important to ensure the system’s reliable and continuous operation. At the European XFEL, there are three photon beamlines accompanied by a 3 km-long vacuum system in total (Dommach *et al.*, 2021 ▸). Different types of vacuum pumps are used to reduce the pressure within each beamline and, once the desired pressure is achieved, SIPs (Schulz, 1999 ▸) take over to maintain a constant vacuum level. In total, 300 SIPs are strategically placed at various locations along all three beamlines.

SIPs refer to a particular type of vacuum pump that operates by ionizing gas molecules through a combination of electric and magnetic fields within so-called Penning cells. The resulting ions are then accelerated toward the pump surfaces (cathode), where getterable gases are chemically pumped and noble gases are buried into the pump material. It helps maintaining a low-pressure environment by continuously pumping out residual gas molecules and preventing them from returning to the chamber. At the European XFEL, these pumps are typically operated at a constant voltage of 3 kV, except during the first few days of commissioning, when voltages of up to 7 kV are applied. During operation, the pumps may be exposed to several noble gases, including xenon, krypton, and argon, which are used in various diagnostic instruments at the European XFEL.

Over the course of their operational lifespan, SIPs may experience the development of an undesirable effect referred to as the Noble Gas Instability (NGI) (Porcelli *et al.*, 2015 ▸). An NGI occurs when the internal components of the pump become saturated with noble gases and subsequently release these gases back into the vacuum chamber, destabilizing the vacuum. This phenomenon can significantly diminish pumping efficiency and ultimately require the pump to be replaced. For large-scale photon sources like XFEL facilities, replacing a pump requires operational shutdown, and restoring vacuum levels can take several hours to days. This makes it crucial to detect pump malfunctions well in advance before reaching the critical stage. The challenge is to monitor numerous pumps around the clock, which is impractical manually.

A key indicator of NGI is the occurrence of recurrent pressure peaks (Maccarrone *et al.*, 2020 ▸). The rate at which these peaks appear is influenced by the beamline pressure; lower pressure levels result in less frequent peak occurrence, which makes their detection considerably more difficult in ultra-high-vacuum conditions. Additionally, pressure peaks can originate from various other sources, including adjustments made by operators to certain parameters.

**Figure 1:**
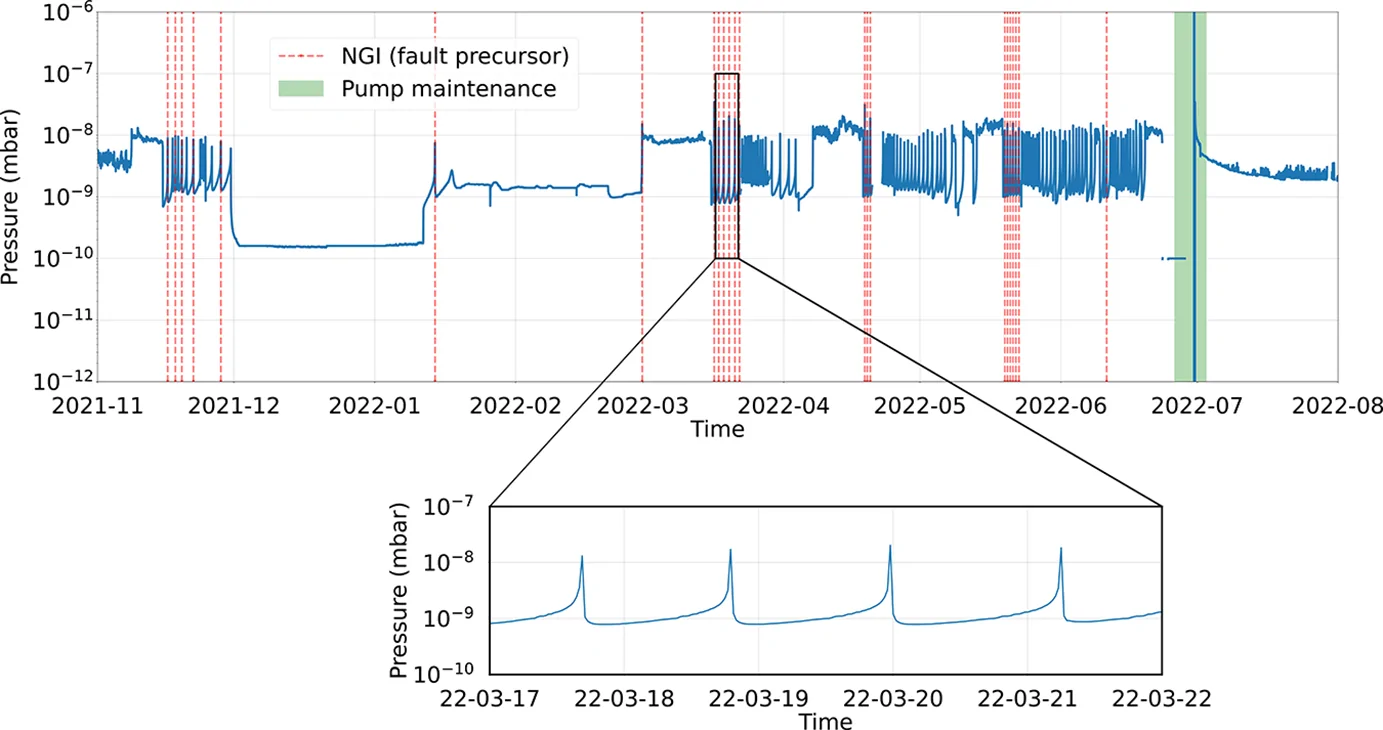
Pressure data obtained from one of the pumps installed at the European XFEL, showing the progression of NGI events (fault precursors) over time. The measurements are averaged over a 30 min window for better visualization. Red markers represent NGI events, while the shaded green region indicates maintenance period. The inset plot shows a magnified view of five days of pressure data, illustrating four NGI instances.

Accurately discerning whether these peaks are attributed to NGI requires domain knowledge and expertise. Currently, this determination relies on manual inspection by vacuum experts, who evaluate various factors such as shape of the peak, operating pressure, pump age, and proximity to noble gas sources in the beamline. As this effect develops gradually, small changes in pressure are difficult to notice, especially during its early stages. Fig. 1 ▸ displays the pressure readings of a SIP over a year, indicating the gradual onset of NGI, which began to show signs in mid-November 2021. At this point, it was not easy to identify this issue just by looking at the data compared with previous months, which also included small fluctuations. Following its initial appearance, only two instances of these peaks occurred, in January and February 2022. Subsequently, it reappeared in March 2022 with increased severity. By June 2022, the issue reached a critical point that required pump replacement. These unnecessary pressure fluctuations may lead to beam instability, and it is crucial to understand the origin of the problem to prevent future issues. Identifying NGI-related patterns in pressure signals and differentiating them from other unrelated fluctuations pose a significant challenge, demanding an automated system capable of precisely detecting and classifying these patterns. Leveraging ML may provide a viable solution for automating the detection of such patterns in pressure data.

## 3. Data

### 3.1. Data collection and annotation

**Figure 2:**
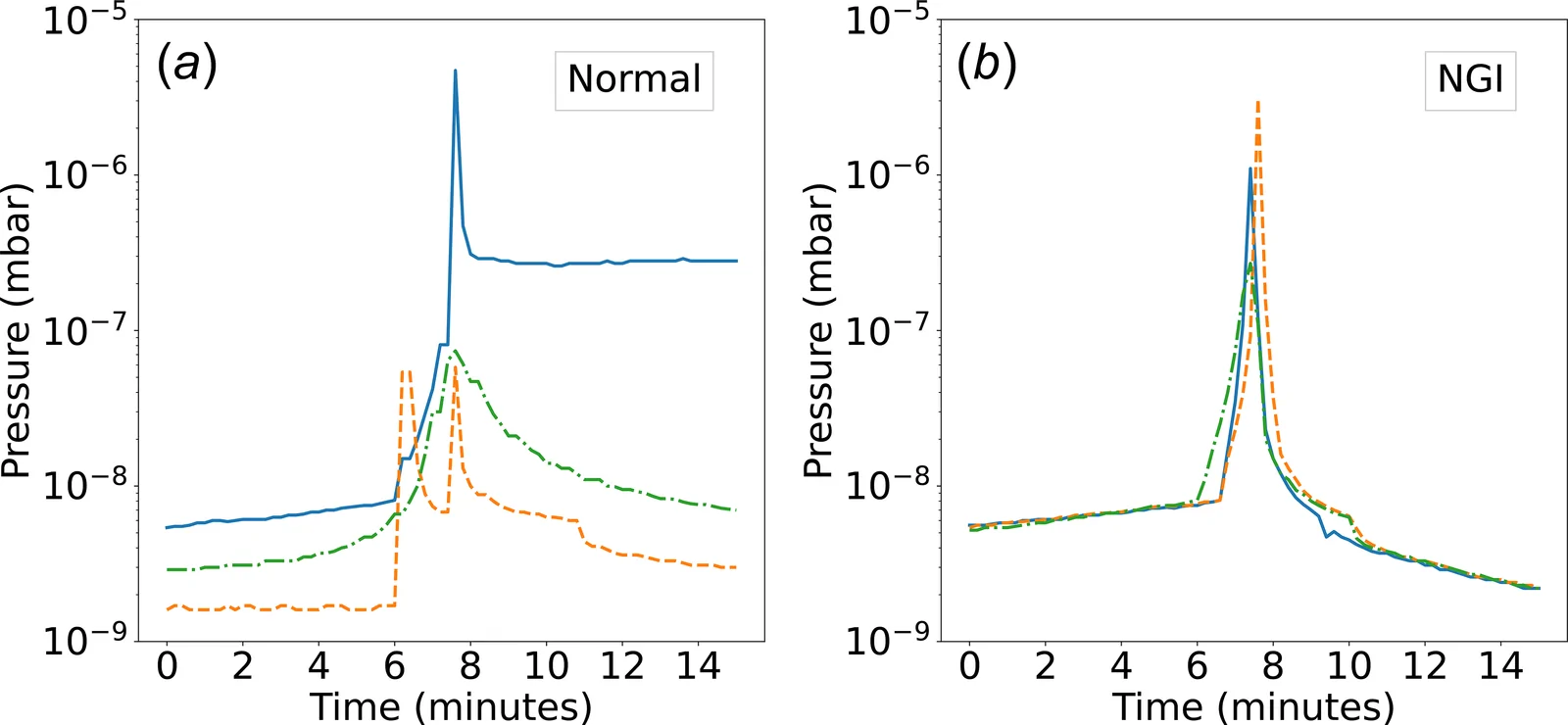
Pressure data examples from the two categories Normal and NGI. Each plot is a 15 min pressure measurement with sampling interval of 12 s (76 points). (*a*) Three examples categorized as Normal, exhibiting intermittent surges often resulting from deliberate adjustments in operational settings, such as the ones mentioned in Appendix A2[App appa]. (*b*) NGI events, characterized by gradual rise and decay of pressure.

The photon beamlines and equipment are controlled through the supervisory control and data acquisition software *Karabo* (Hauf *et al.*, 2024 ▸), which also features an integrated data logging and database management system for storing historical data related to all process variables. We examined historical data from multiple pumps installed at various physical locations. Among them, two pumps (denoted as **p1** and **p2** in this work) had exhibited NGI effects, while the rest remained in a healthy state. To develop a meaningful dataset, we first conducted a manual inspection of the trends in pressure measurements over time (see Appendix A1[App appa]). Consequently, we extracted a 15 min window from the pressure time series to use as a data sample 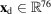. We gathered instances displaying NGI events and many other examples to capture the diverse trends present in pressure data corresponding to different operational states of the system. Consequently, we obtained a manually labeled dataset having 115 examples corresponding to NGI, and 14927 instances representing the normal state denoted as Normal. Some examples from both categories are shown in Fig. 2 ▸.

### 3.2. Feature engineering

**Figure 3:**
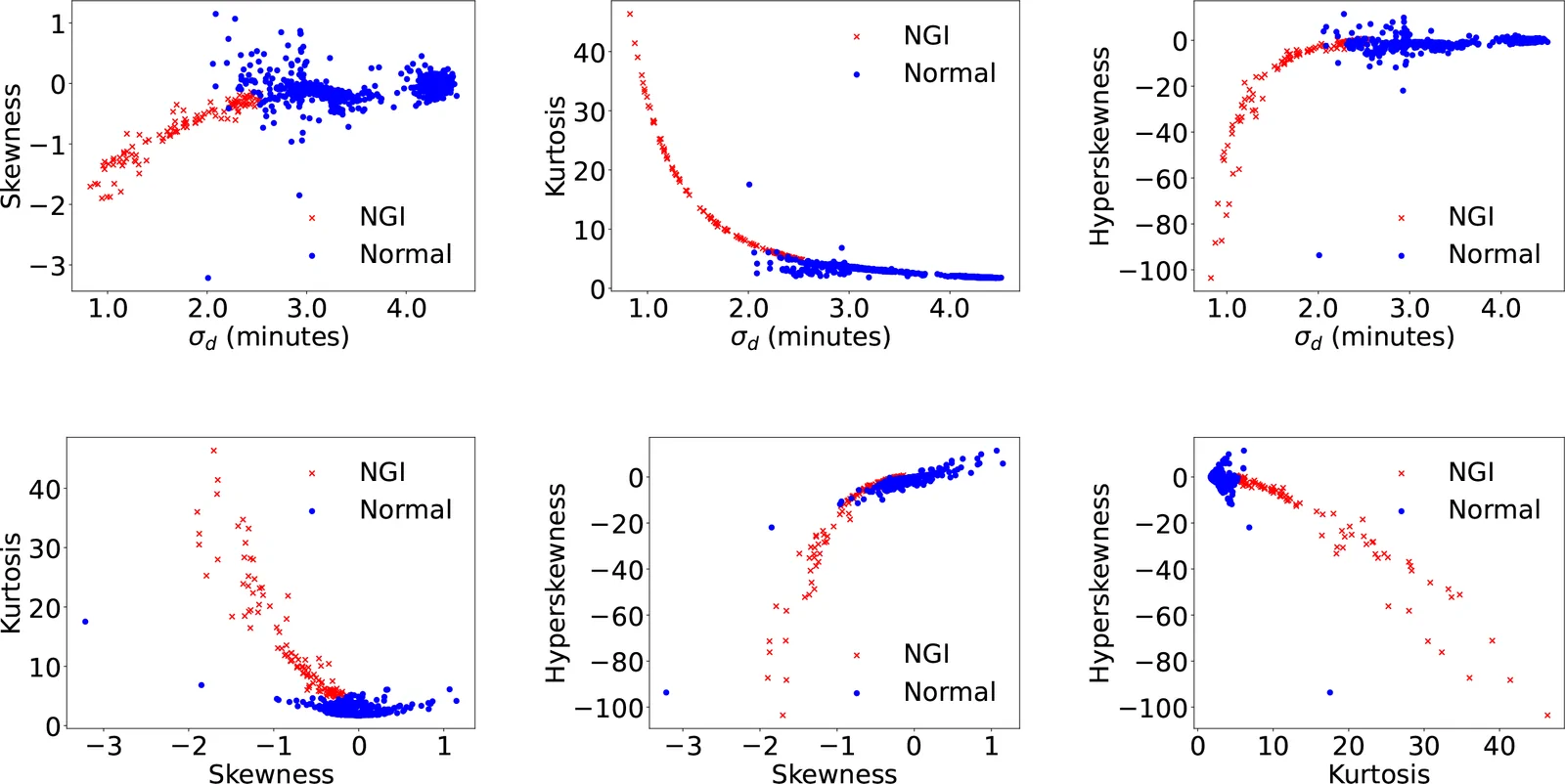
Visualization of the features distribution: red points represent instances of NGI, while the blue points are samples from the Normal category.

Feature extraction is a crucial step that involves identifying and capturing important patterns within the data. It requires converting raw signals into a compact format while keeping essential information. Subsequently, these extracted features can undergo further analysis using ML algorithms. Our dataset, as described in Section 3.1, comprises time series data characterized by peaks of varying shapes. Consultation with vacuum experts revealed that pressure measurements corresponding to NGI events exhibit a gradual rise and decay, in contrast to other types of pressure spikes, which typically appear as sudden, sharp increases with distinct subsequent trends, as shown in Fig. 2 ▸. To effectively capture these distinctive temporal patterns, we extracted statistical features, namely standard deviation (σ_d_), skewness, kurtosis and hyperskewness from the pressure time series, as defined in Appendix *B*[App appb]. After applying these transformations, each data sample 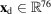 was converted into a four-dimensional vector 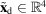 in feature space. Fig. 3 ▸ shows scatter plots of the features computed for all data samples. These features were subsequently used to train the SVM classifier described in Section 4.1.

### 3.3. Data imbalance

**Figure 4:**
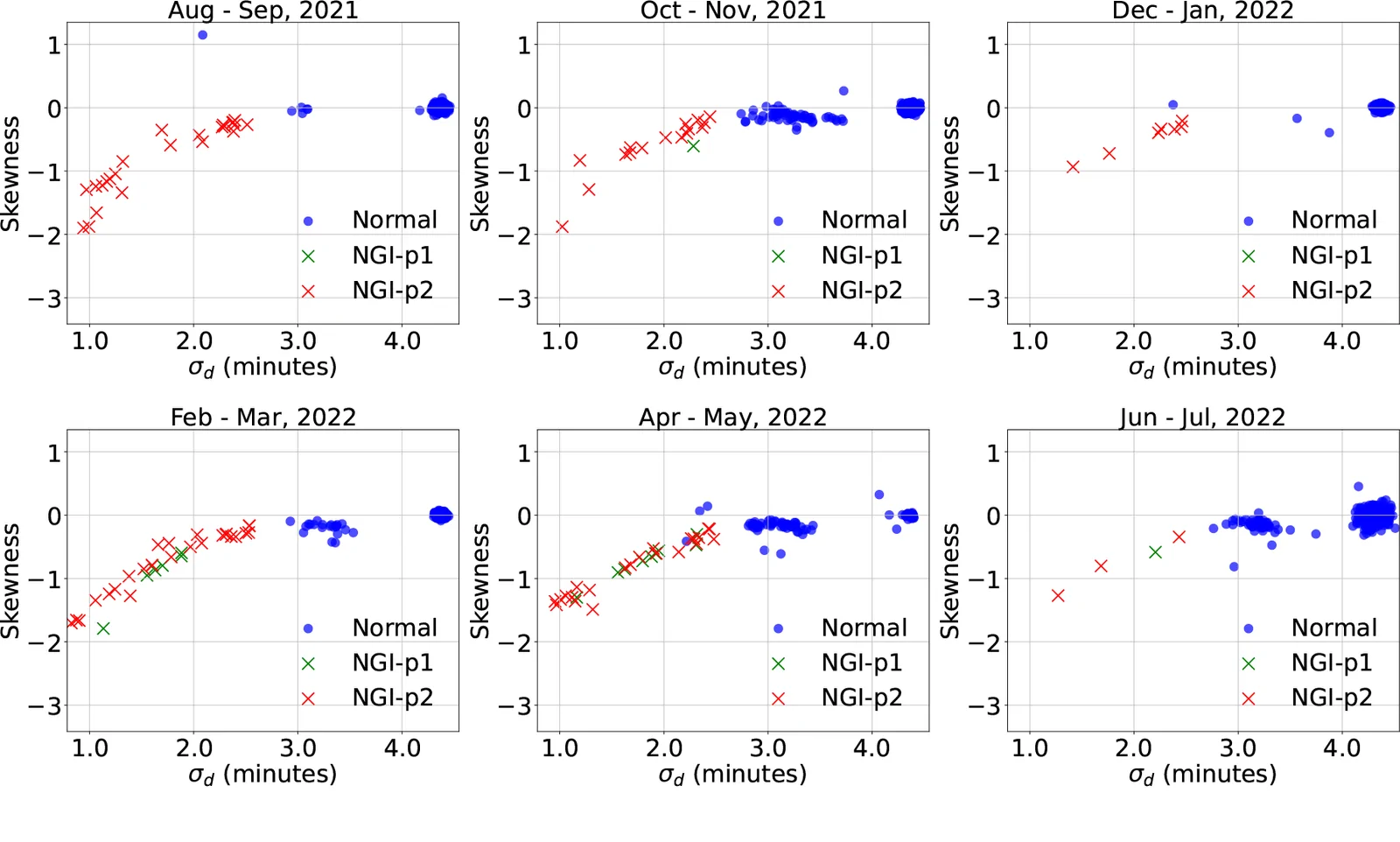
Feature distribution (standard deviation versus skewness) corresponding to NGI occurrences over a one-year time-frame, grouped into two-month intervals. **p1** and **p2** correspond to two SIPs.

We analyzed data in feature space to assess potential drifts over time. Fig. 4 ▸ illustrates the distribution of two features—standard deviation and skewness—over a one-year period, grouped into two-month intervals. NGI examples were drawn from two pumps, **p1** and **p2**, while Normal instances originated from multiple other pumps.

In general, no significant drifts were observed over time for these features. As can be observed, NGI examples from **p1** were sparse, limiting the ability to capture the full range of behavior associated with NGI. This sparsity reflects the inherent class imbalance common in practical systems, where the available data are often insufficient to comprehensively characterize all minority class patterns.

Under-representation of minority class samples can lead to a shift in the data distribution after deployment, when the model is exposed to previously unseen examples or it is transferred to a new hardware setup. Furthermore, when trained on imbalanced datasets, classifier models often fail to accurately identify the minority class, yielding results comparable with those of a naive model that always predicts the majority class (Cortes *et al.*, 2025 ▸). To address data imbalance, we have investigated two different techniques: cost-sensitive learning and SMOTE, as described in Section 4.

Cost-sensitive approaches (He & Garcia, 2009 ▸) work by assigning higher penalties when samples from the minority class are misclassified, thus reducing the model’s bias toward the majority class. On the other hand, SMOTE balances the dataset and generates new synthetic samples by interpolating between existing minority class instances.

### 3.4. Dataset preparation

To evaluate the performance of ML-based solutions under such conditions, we constructed four datasets using different combinations of the collected data:

(i) DS-I: all collected data samples were randomly split into training and test sets with a 70:30 ratio, while maintaining the proportion of NGI examples.

(ii) DS-II: NGI examples were partitioned with respect to the two pumps: **p1** data were used for training, and **p2** data for testing (while Normal examples were randomly split across training and test sets). With this, we aim to investigate how the ML model generalizes with respect to different pumps.

(iii) DS-III: same as DS-II, but with the pump allocation reversed—**p2** data for training and **p1** for testing.

(iv) DS-IV: NGI examples were divided into training and test sets based on occurrence time. The training set comprised NGI examples from **p2** recorded in August 2021, whereas the test set included examples from pumps **p1** and **p2** recorded between September 2021 and July 2022. This temporal split was performed to assess the ML model’s ability to generalize from early- to later-stage NGI events. Normal class examples were randomly split into training and test sets.

Table 1 ▸ presents an overview of prepared datasets.

## 4. Model development

This section provides an overview of the ML models employed in this study, namely support vector machines and convolutional neural networks. Additionally, Section 4.3 discusses the synthetic minority over-sampling technique which is utilized to mitigate the effects of class imbalance in ML datasets.

### 4.1. Support vector machine

SVM is a powerful supervised learning model used for classification and regression tasks. SVM works by finding the optimal hyperplane in a high-dimensional space that best separates different classes while maximizing the margin between the classes (Salcedo-Sanz *et al.*, 2014 ▸). This margin is the distance between the hyperplane and the nearest data sample (support vectors), allowing SVM to generalize well to unseen data as in our case. Consider a dataset 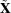 with *D* training data samples. Each data sample is represented as 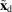 where 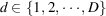 having binary class label *y*_*d*_ ∈ {−1, 1}. The objective of SVM is to find the hyperplane 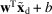 = 0 that should classify the data samples, subject to 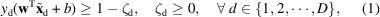
where ζ_*d*_ are slack variables allowing for misclassifications. The optimization problem is formulated as follows,
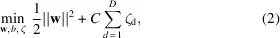
where **w** is the weight vector perpendicular to the hyperplane, *b* is the bias term, ||…|| represents the Euclidean norm of the quantity inside, and *C* is a regularization parameter controlling the trade-off between margin maximization and classification error. Margin is the distance between the decision boundary and the nearest data points from each class, defined by two parallel hyperplanes, 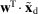 + *b* = 1 and 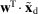 + *b* = −1. Points lying on or within these boundaries are known as support vectors, as they fully determine the position of the decision boundary.

For imbalanced datasets, standard SVM tends to bias the decision boundary toward the majority class, leading to poor performance on the minority class (He & Garcia, 2009 ▸). To address this challenge, a commonly employed strategy is to perform cost-sensitive learning and incorporate class-dependent weights into the objective function,
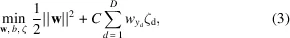
where 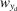 is the weight of sample having class label *y*_d_, computed as
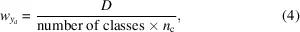
where *n*_c_ is number of points in the respective class.

To handle non-linearly separable data, SVM utilizes kernel functions that implicitly map the data into a higher-dimensional space where linear separation is possible. Common kernels include polynomial, Gaussian radial basis function (RBF), and sigmoid (Vapnik, 1995 ▸). In our study, we employed SVM with balanced class weights using three kernels: linear, polynomial, and RBF.

### 4.2. Convolutional neural networks

CNNs (O’Shea & Nash, 2015 ▸) utilize convolutional layers that perform convolutions on input data. This involves sliding a small filter across the input data, performing element-wise multiplications, and summing the results. In the context of time series classification, a CNN’s architecture typically begins with input data structured as a series of sequential observations. The initial layers of the CNN learn low-level features, such as edges or basic patterns, through convolutions. As the network progresses, the higher layers learn more abstract and complex representations based on these low-level features. The mathematics underlying CNNs involves the convolution operation itself, employing techniques such as padding and stride to control the output size. During the convolution process, the filter moves across the subsets of the input data. At each step, it computes the dot product between the filter and the corresponding input subset. This is crucial for feature extraction, enabling the network to identify relevant patterns within the time series data. Moreover, CNNs for time series classification often incorporate pooling layers, such as max-pooling, to downsample the extracted features, reducing computational complexity and retaining the most important information. The final layers of the network typically involve fully connected layers and Softmax activation for classification, where the network maps the learned features to different classes, providing a probability distribution over the possible outcomes.

**Figure 5:**
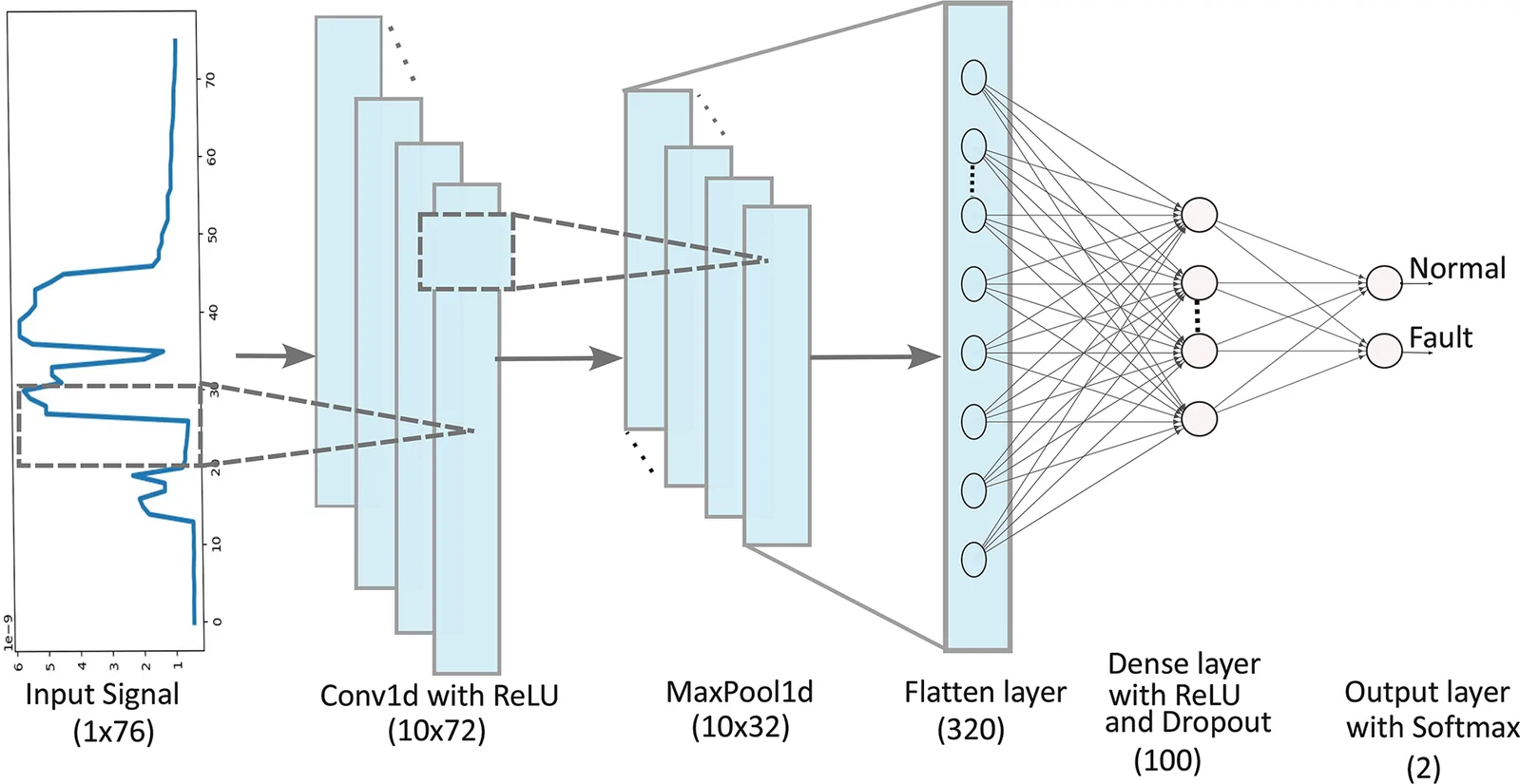
Schematic of the CNN model architecture used for binary classification.

CNN classifier architecture, used in this study, is illustrated in Fig. 5 ▸. The model processes input data structured as a 1D tensor of shape (1, 76), producing a binary probabilistic output that classifies each sample as either Normal or NGI. The architecture consists of (i) a Conv1D layer (10 filters, kernel size 5) with ReLU activation, followed by max-pooling (size 10), (ii) a flattened layer followed by two dense layers (320 and 100 nodes, both ReLU-activated), with a 20% dropout layer, and (iii) a final two-node dense layer with Softmax activation.

We used cost-sensitive learning in our CNN model as well to address the problem of imbalanced data. A weighted cross-entropy loss function was used, with class weights inversely proportional to the number of samples available for each class, thereby encouraging the model to place greater emphasis on minority class.

### 4.3. The synthetic minority over-sampling technique

SMOTE (Chawla *et al.*, 2002 ▸) is a popular method used to address imbalanced datasets in supervised learning tasks. It works by generating synthetic examples for the minority class rather than simply duplicating existing ones. This is achieved by interpolating between existing minority class instances, which helps to balance the class distribution and improve the performance of classification algorithms. We applied SMOTE to the minority class, increasing its size to 50% of the majority class. Oversampling was performed in the feature space after feature extraction, and the generated data were used to train the SVM model with cost-sensitive learning, as detailed in Section 4.1.

## 5. Results and discussion

**Figure 6:**
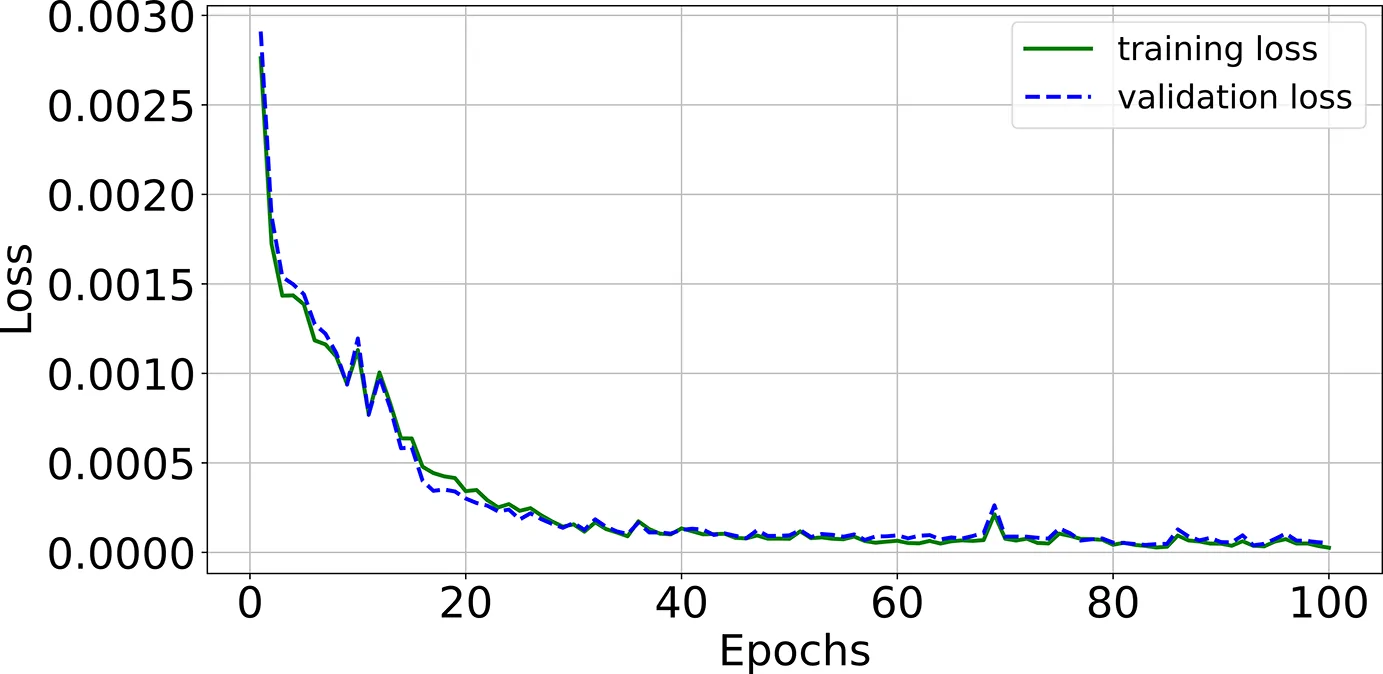
Training and validation loss across epochs during CNN training for DS-I.

We conducted multiple experiments to compare the performance of the ML models described in Section 4, applied to four different data combinations outlined in Table 1 ▸. The training data were normalized using Z-score standardization, which ensures zero mean and unit variance. The resulting normalization parameters were subsequently applied to the test set to avoid data leakage. Normalized training data were used to train four different classifier models: (*a*) SVM with a linear kernel, (*b*) SVM with an RBF kernel, (*c*) SVM with a polynomial kernel, and (*d*) CNN. For SVM classifiers, data samples were first transformed into feature vectors as described in Section 3.2. In contrast, for the CNN, time series data were fed directly into the input layer after normalization. In this case, the training set was further divided into two subsets: Group A (70%) for training the model, and Group B (30%) to validate performance during training. Fig. 6 ▸ illustrates the average loss per batch during the training process across 100 epochs. We used the *Adam* (Kingma & Ba, 2014 ▸) optimizer with a learning rate of 0.001 and a batch size of 100.

To address the issue of class imbalance, we applied cost-sensitive learning by assigning class weights proportional to their respective class ratios within the cost function for both SVM and CNN, as described in Sections 4.1 and 4.2, respectively. Furthermore, we explored the utilization of SMOTE in combination with cost-sensitive SVM to evaluate which approach better handles imbalanced data. SMOTE was employed to generate synthetic samples for the NGI class, using feature domain data from the training set only, with a 50% oversampling ratio. Fig. 9(*c*) shows synthetic samples along with training data. These augmented samples along with the original samples in the training set were subsequently used to train SVM models with all three kernel types.

**Figure 7:**
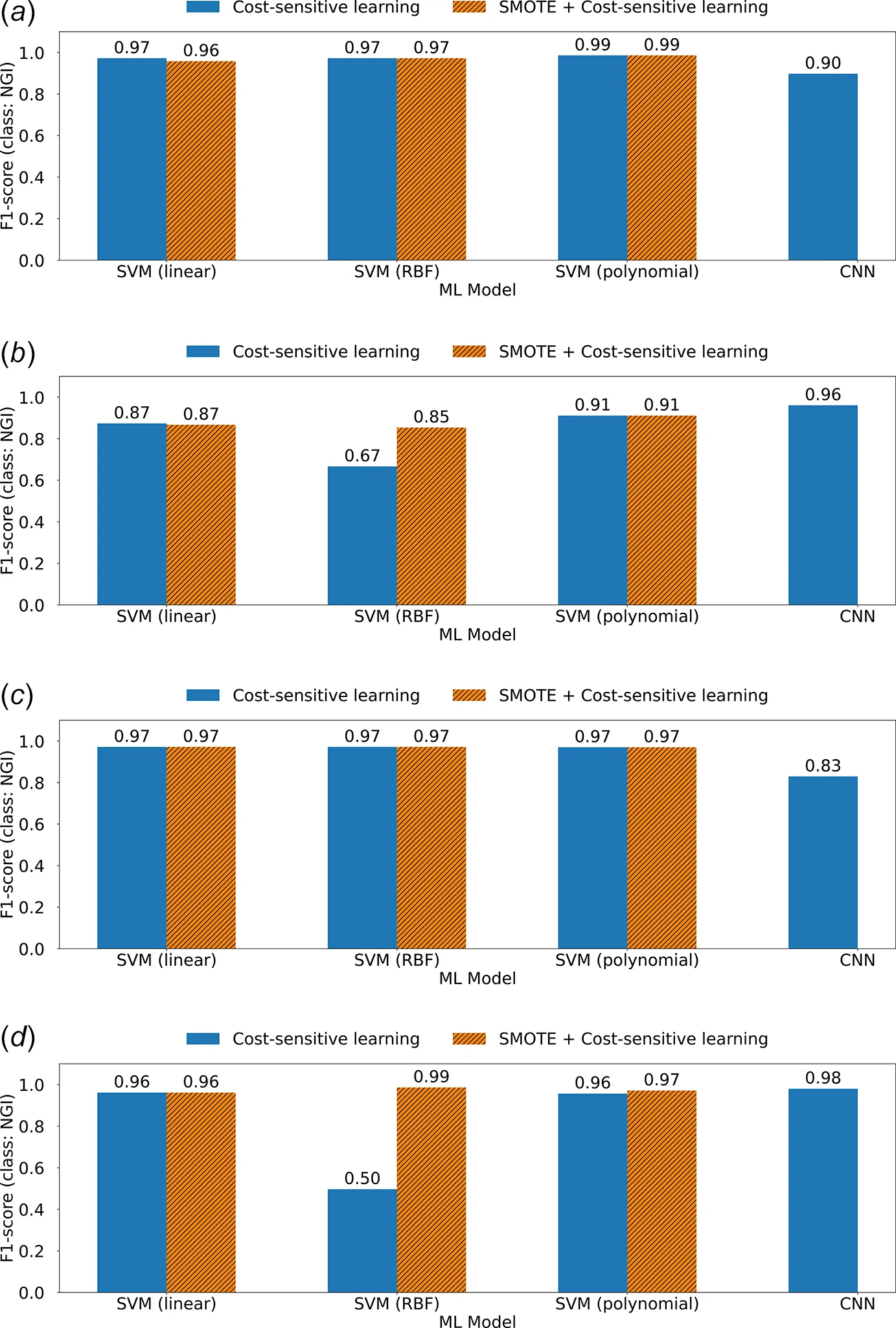
Performance evaluation: comparison of ML models based on F1-scores of the minority class (NGI). (*a*) DS-I, (*b*) DS-II, (*c*) DS-III, and (*d*) DS-IV (*cf.* Table 1 ▸).

The performance of all ML models was evaluated using the designated test set. To provide a comprehensive assessment in the context of imbalanced data, we focused on the precision, recall, and F1-score of the minority class (NGI) as evaluation metrics (see details in Appendix *C*[App appc]). Fig. 7 ▸ shows the classification results in terms of F1-score, while precision and recall values are reported in Table 3 in Appendix *D*[App appd].

### 5.1. Validation on new data after deployment

After completing model training and validating performance on the held-out test set, we deployed ML models to detect NGI events in unseen data. These new data were collected from 54 additional pumps over a continuous three-year period, from August 2022 to August 2025. In addition, data from a small number of pumps collected before August 2022 were available and included to construct a comprehensive evaluation dataset. To identify peaks in the pressure time-series, we detected local maxima where the pressure exceeded 2 × 10^−9^ mbar. The *find_peaks* function from the *SciPy* (Virtanen *et al.*, 2020 ▸) signal processing toolbox was used for this task. For each detected peak, a 15 min segment was extracted yielding 1126034 candidate samples.

**Figure 8:**
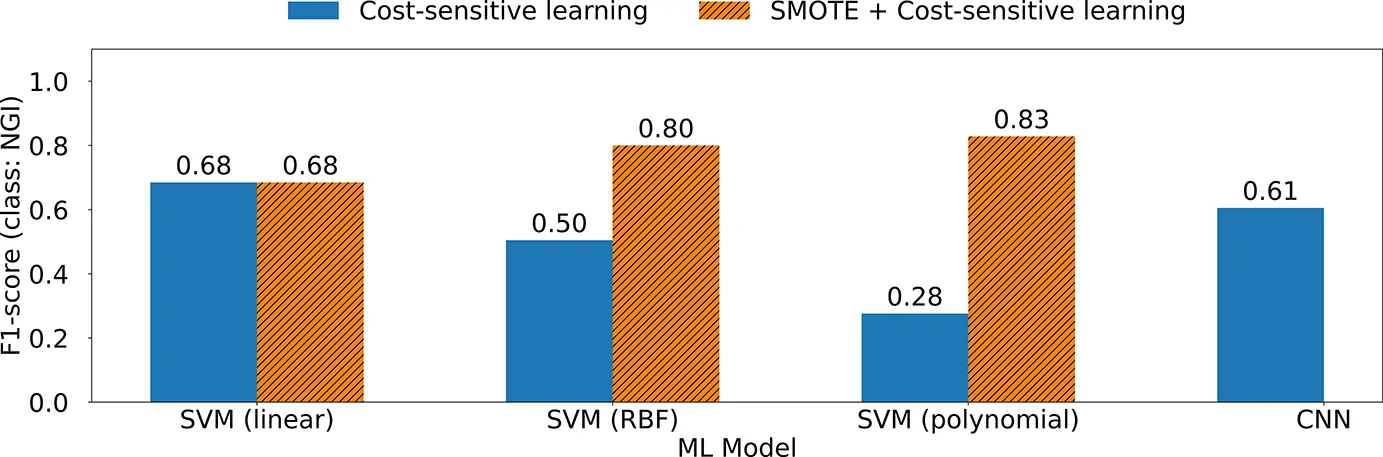
Post-deployment performance evaluation: comparison of ML models based on F1-scores of the minority class (NGI).

These instances were then analyzed by the deployed models to detect potential NGI occurrences. The performance of the models was assessed using the standard precision metric defined in equation (12). While precision could be directly computed from the model predictions, recall required additional ground-truth labels. However, manually reviewing and labeling all 1126034 samples was not feasible due to the large dataset size. Instead, to estimate recall, we constructed a labeled subset of this new data. This subset included all events detected as NGI by all the models (both *True positives* and *False positives*). Moreover, to select a representative subset from the rest of the deployment data, we clustered the data samples into *k* = 1000 clusters using the *k*-means algorithm (Lloyd, 1982 ▸) and selected samples closer to the centroid of each cluster. Overall, this new subset included 46 true NGI events and 963 Normal events. Using this curated subset, we computed recall and F1-score for each deployed model. This approach provided a balanced evaluation framework: precision was derived from the full dataset, while recall and F1-score were estimated from the manually labeled subset. Together, these metrics offered a comprehensive view of model performance under real-world deployment conditions. The results are presented in Fig. 8 ▸ in terms of the F1-score of the minority class, while precision and recall are reported in Table 4 in Appendix *D*[App appd].

### 5.2. Discussion

#### 5.2.1. Handling imbalanced data

**Figure 9:**
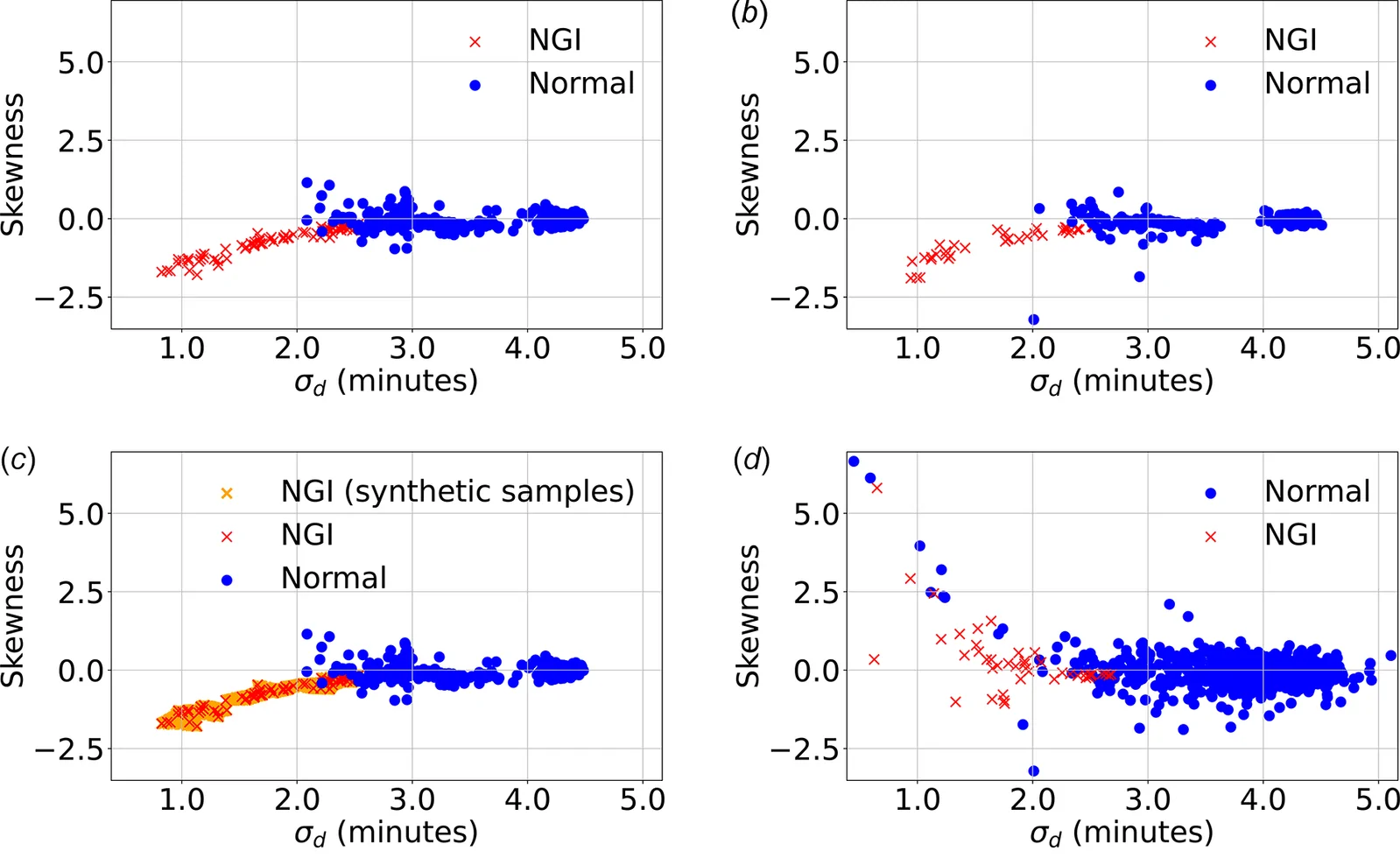
Visualization of data in feature space (standard deviation versus skewness). Panels (*a*) and (*b*) represent the training and test sets for DS-I, respectively. Both show similar distributions of samples in the feature space, as the training and test sets were randomly divided while maintaining the class ratio. Panel (*c*) displays the training data along with synthetic samples created using SMOTE. Panel (*d*) illustrates the distribution of new data after deployment, which shows a significant shift in distribution of NGI examples.

For DS-I, SVM with all kernel methods demonstrated strong and comparable performance for the test sets, with polynomial kernel achieving the best performance (*cf.* Fig. 7 ▸ and Table 3). Figs. 9 ▸(*a*) and 9(*b*) illustrate the training and test sets for DS-I, respectively, both of which exhibit similar distributions of data samples, thereby contributing to the strong performance observed across all methods.

However, for DS-II, as mentioned in Section 3.4, examples only from **p1** were used to train the models. These training examples were fairly sparse compared with the test set examples from **p2**, leading to an overall decrease in the performance of SVM models. Notably, for the SVM model with an RBF kernel, the combination of SMOTE and cost-sensitive learning outperformed cost-sensitive learning alone. Similarly, for DS-IV, all models showed good performance, apart from the SVM with RBF kernel trained using cost-sensitive learning alone. As mentioned in Section 3.4, this dataset was compiled to test the performance of ML models when trained using only few NGI examples occurring at an early stage of a pump’s life-cycle. The overall good performance suggests that models can generalize from early- to later-stage NGI events.

The poor performance of SVM with RBF kernel using cost-sensitive learning, in the case of DS-II and DS-IV, could be due to the limited number of minority class samples in the training data. This may be due to the fact that focusing solely on balancing classification costs in SVM can cause the model to overfit to the available minority class samples, thereby reducing its ability to generalize well to unseen data. This issue is especially prominent if the minority class samples are not sufficiently representative or fail to encompass the full range of NGI cases (as in the case of DS-II). Therefore, relying exclusively on cost-sensitive learning might not be an optimal strategy. This suggests that integrating data augmentation techniques like SMOTE with cost-sensitive methods can be more effective for handling imbalanced datasets.

These observations persist even in the deployment dataset (*cf.* Fig. 8 ▸ and Table 4). After deployment, we observed a significant shift in the distribution of NGI examples when examining the feature space. Fig. 9 ▸(*d*) shows the selected subset of data after deployment. Specifically, the NGI samples are now positioned quite far from their original distribution. This caused a decline in model performance after deployment. However, even in this scenario, the combination of SMOTE with cost-sensitive learning of SVM (RBF and polynomial kernels) demonstrates reasonably good performance (an F1-score of 0.80) in identifying new NGI events.

The better performance of this method can be attributed to the ability of SMOTE to create synthetic samples by interpolating between existing minority class examples, resulting in more diverse and representative training data. By filling in the feature space between minority samples, SMOTE makes the minority class distribution more continuous and helps classifiers learn smoother decision boundaries, thus reducing bias toward the majority class. Consequently, SMOTE provides additional relevant samples, allowing the model to delineate broader decision regions, particularly useful in handling imbalanced datasets, specifically when limited samples hinder comprehensive coverage of minority class regions in feature space. In contrast, cost-sensitive learning relies solely on the limited information available from existing examples. However, SMOTE may not always outperform in all scenarios as it can generate noisy or overlapping samples, particularly when classes are not well separated. For scenarios with significant overlap, variations such as Borderline SMOTE could be considered to address these limitations. However, in our data, overlap issues are not as significant as the shift in minority class regions in feature space, which may explain why SMOTE performs better in this context.

#### 5.2.2. Comparison between SVM and CNN

Regarding the comparison between CNN and SVM, we observe that both methods achieved strong results for DS-I, DS-II and DS-IV but the CNN’s performance declined for DS-III with a higher number of false alerts (*cf.* Fig. 7 ▸, and Table 3 in Appendix *D*). After deployment, CNNs exhibited superior generalization to previously unseen data under distributional shift, outperforming the SVM with cost-sensitive learning (*cf.* Fig. 8 ▸, and Table 4 in Appendix *D*). However, its effectiveness remained lower than that of SVM combined with SMOTE and cost-sensitive learning, suggesting that this integrated approach provides a more robust solution.

Moreover, it is important to highlight that CNNs automatically learn features directly from raw input data, reducing the need for manual feature engineering as compared with SVM in which input features need to be carefully crafted by domain experts, and the quality of features is crucial for the performance. CNNs are easier to use, but feature engineering coupled with SVM is more interpretable in terms of understanding how and why the model makes certain predictions. Interpretability plays a pivotal role, especially in the context of large-scale scientific facilities, where end users are from different scientific backgrounds. Interpretability is valuable not only during the initial stages of data exploration and visualization but also in operational settings, where it helps explain false alerts and facilitates the incorporation of the feedback from domain experts.

#### 5.2.3. NGI detection performance across individual pumps

To assess how well the model, trained using NGI examples from two specific SIPs, generalizes to different SIPs, we employed it to 54 previously unseen pumps, as described in Section 5.1. Vacuum experts confirmed that six of these pumps exhibited the NGI effect.

In particular, we analyzed the predictions of the best-performing model, the SVM with a polynomial kernel trained using the combination of SMOTE and cost-sensitive learning, to examine how NGI detections were distributed across individual pumps. The model flagged 53 instances as potential NGI events, with detections occurring in 13 of the 54 pumps. Of these 13 pumps, five were confirmed to have genuinely exhibited the NGI effect, while the remaining eight were associated with false alerts.

**Figure 10:**
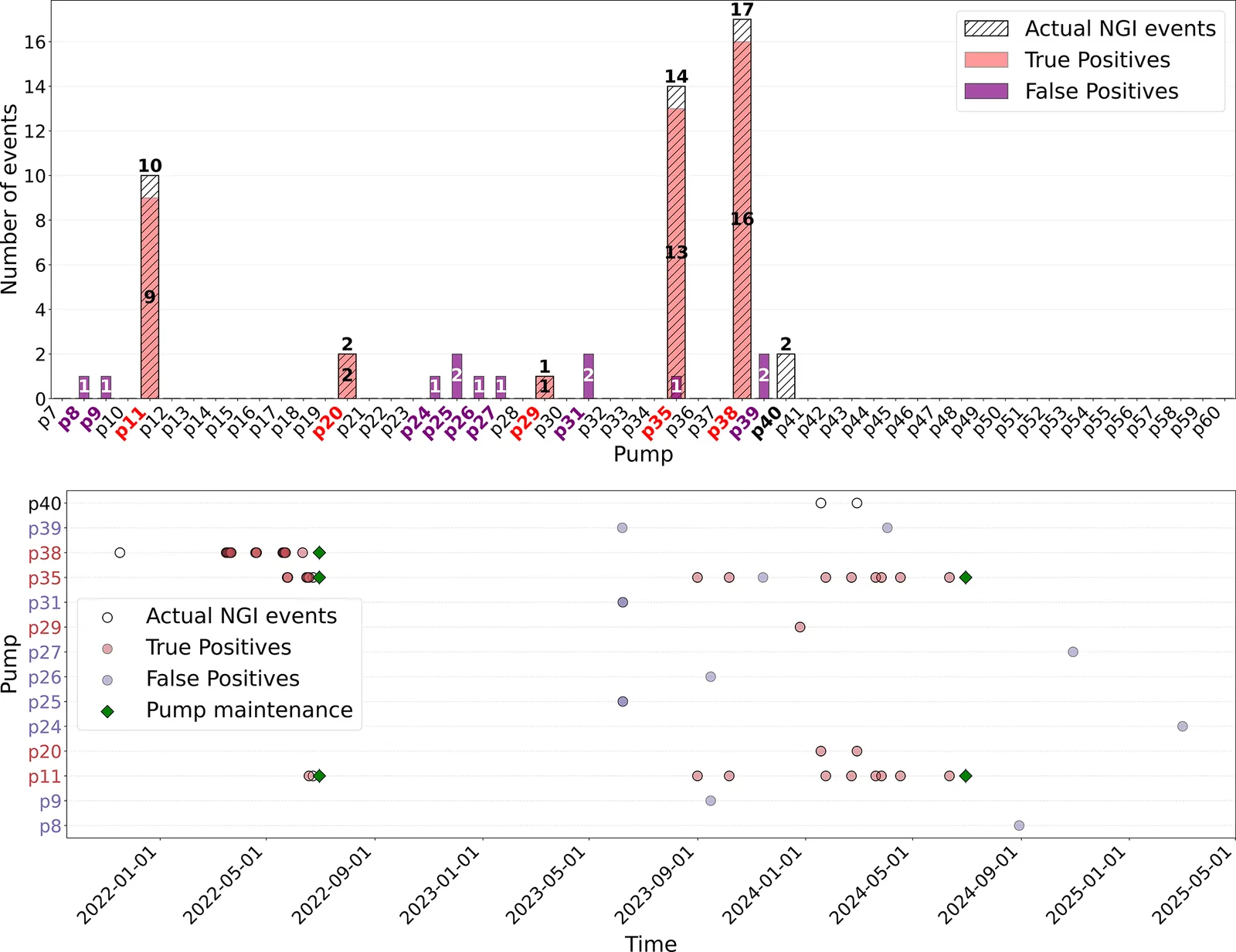
Performance in individual pumps. (*a*) Distribution of actual and model-detected NGI events across individual pumps. Model detections classified as True Positives correspond to correctly detected NGI events, while False Positives correspond to events incorrectly classified by the model as NGI. (*b*) Temporal distribution of True Positives, and False Positives across pumps.

Fig. 10 ▸(*a*) compares the model’s detections with the confirmed NGI events for each pump, while Fig. 10 ▸(*b*) shows how these events are distributed over time. In both figures, ‘Actual NGI events’ refer to NGI events confirmed by experts within the evaluation subset, ‘True Positives’ refer to actual NGI events correctly detected by the model, and *False Positives* refer to instances from Normal category incorrectly classified as NGI. It is important to highlight that, when an NGI related pressure peak occurs in one pump, a similar peak, with a delay of a few seconds may sometimes also be observed in a neighboring pump, as can be seen in the case of **p11** and **p35** in Fig. 10 ▸(*b*). For evaluation purposes, such secondary responses were also labeled as NGI, as they may still contain useful information related to NGI activity.

The frequency and temporal distribution of detected events may provide additional information to identify false alerts. In fact, as can be noticed, the majority of the detections belonged to the three pumps **p11**, **p35** and **p38** [Fig. 10 ▸(*a*)]. In contrast, pumps associated with false alerts typically contained only one or two detected events over the entire evaluation period. This difference suggests that repeated events affecting the same pump may provide useful insight to distinguish between genuine NGI events and False Positives. However, the two other pumps (**p20** and **p29**) also showed confirmed NGI events, though at lower counts of 2 and 1, respectively.

**Figure 13:**
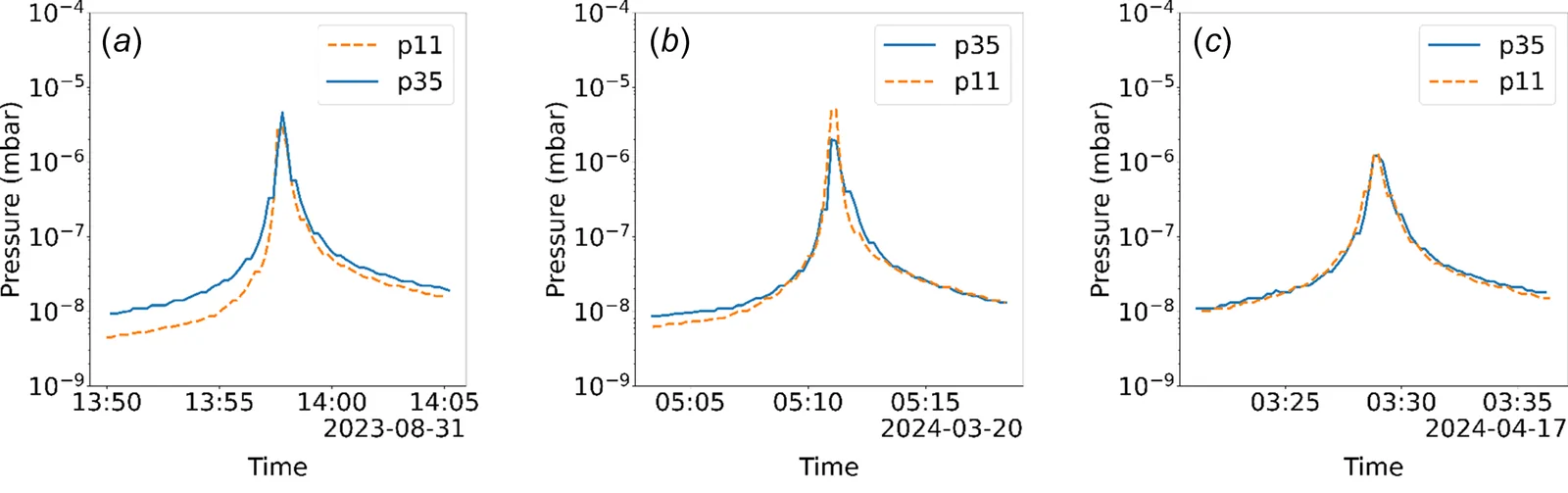
Examples of detected pressure peaks in neighboring pumps **p11** and **p35**, occurring within a few seconds of each other. (*a*) The primary peak occurred in **p35**, with a secondary response observed in **p11**. (*b*) The reverse case, where the primary peak occurred in **p11** and the secondary response was observed in **p35**. (*c*) An ambiguous case where the similar peak amplitudes made it difficult to identify the originating pump.

Furthermore, a strong temporal correlation between events detected in pumps **p11** and **p35** can also be noticed. The two pumps are physically close, and as such pressure measurements in both pumps are affected by their neighboring SIP. In such cases, vacuum experts identify the originating pump by manually inspecting the shape of the two peaks and comparing the corresponding amplitudes. For events detected in **p11** and **p35**, experts concluded that both pumps exhibited NGI effects, as the pump with the higher peak amplitude varied across events (see Appendix D1[App appd], in particular Fig. 13).

Moreover, for three of the five pump maintenance events [as shown in Fig. 10 ▸(*b*)], the model began detecting NGI events, on average, approximately 200 days before pump replacement. From a practical perspective, these results suggest that the model can serve as a diagnostic aid for vacuum experts. However, improving its robustness with respect to detection frequency and distinguishing primary NGI events from secondary responses in neighboring SIPs remain important topics for future research.

#### 5.2.4. Improving post-deployment performance

As detailed in Section 5.2.1, model performance declined following deployment. Specifically, the best performing model, SVM combined with SMOTE, experienced a decrease in the F1-score for the NGI class from 0.91 to 0.83. This reduction in performance can be attributed to the model’s limited ability to generalize to previously unseen data, specifically due to the imbalanced nature and under-representation of the minority class in the original training data. Such challenges are common when ML models are deployed in real-world applications. As a result, these models may require adaptation and retraining. To better understand and address the observed performance degradation, we focused on data instances situated within the SVM’s margins. In principle, a well trained SVM maximizes the margin between classes by constructing a decision boundary that optimally separates the data (see Section 4.1). However, if an increasing number of cases are found within the margins after deployment, this indicates that the model is encountering previously unrepresented examples, suggesting a need for boundary adjustment and retraining.

To enhance the reliability of our fault prediction system during deployment, we partition the data 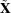 into two mutually exclusive subsets 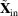 and 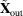, such that 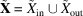, where 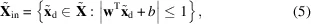

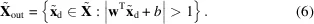
Here, 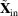 denotes the data samples located inside the margins of the SVM, while 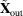 denotes the samples outside the margins. Equations (5) and (6) hold true for SVM with a linear kernel, while, for RBF and polynomial kernels, data samples located inside the margin can be identified via the kernel method (Vapnik, 1995 ▸).

We propose that the decision of the ML model should be trusted only for incoming data points that closely resemble the training distribution, specifically for samples in 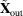. For new or potentially ambiguous examples, particularly those within the SVM margins, we mark these cases as ‘Inconclusive’ and defer them to domain experts for manual inspection and classification. Subsequently, we recalculate the F1-score for samples only in 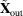, and assess the cost implications of this human-in-the-loop approach in terms of the number of samples in 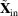 that require manual inspection. The findings are presented in Table 2 ▸. All models showed comparatively better performance for the 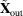 subset. This shows that the method effectively identified data samples that the SVM model tended to misclassify and excluded them from the decision-making process. Specifically, the combination of SMOTE with SVM (RBF kernel) yielded the best F1-score of 0.98, at the cost of manually inspecting 34 samples (out of a total of 1009 samples). Such samples can later be used to retrain the model. This strategy is intended to maintain high classification performance after deployment, while effectively managing the workload for domain experts.

Overall, this work aims to contribute to the detection of NGI related events in pressure data, enabling timely identification of SIPs that have developed the NGI effect. Top performing models, achieving an F1-score of 0.80 and 0.83 after deployment, were based on SMOTE combined with SVM using RBF and polynomial kernel, respectively. The best model detected 53 events of which 41 were related to true NGI events in multiple pumps, whereas 12 were false alerts. Identifying these 41 NGI events using a naive peak-detection approach would have required manual review of 43297 candidate samples (see Appendix *E*[App appe] for details).

However, with our ML-based approach, only 53 samples were needed to be examined, reducing manual inspection efforts by more than 99%. As such, the methodology described in this paper enables a largely automated inspection of pressure events in SIPs. Moreover, by inspecting samples lying inside the margins of SVM, the F1-score can be further increased with a little cost of manually inspecting a few additional samples. These samples, along with the model’s predictions, specifically false alerts, can be used to iteratively retrain the model for continual learning and further improve the model’s accuracy and robustness over time.

## 6. Conclusion

In this work, we present the development of an early fault detection system for Sputter Ion Pumps (SIPs) installed at the European XFEL, with a focus on identifying the noble gas instability effects in highly imbalanced data. Such events, if not detected in a timely manner, can lead to unwanted interruptions and operational downtime of the beamline. Our primary objective is to leverage supervised ML techniques to automatically classify pressure time series data into respective categories. We investigated the application of two widely used ML algorithms, convolutional neural networks (CNNs) and support vector machine (SVM), to analyze the data and compare their performance. Operational measurements were collected from pumps installed at the facility, and a labeled dataset was created with the help of domain experts. For the SVM model, we incorporated a feature extraction method designed to capture the distinctive statistical properties of the time series data.

To address the challenge of significant class imbalance, we investigated two approaches: cost-sensitive learning and the synthetic minority over-sampling technique (SMOTE). Experimental results demonstrated that the combination of SMOTE with cost-sensitive learning in SVM achieved the best performance across various testing scenarios, with the minority class F1-score exceeding 91%.

Post-deployment evaluation on unseen data revealed a distribution shift, which resulted in a decline in performance for models trained using cost-sensitive learning. Nevertheless, the combination of SMOTE and cost-sensitive learning in SVM continued to effectively detect new NGI events, achieving a minority class F1-score of 80%, thereby significantly reducing the manual inspection efforts.

An advantage of the developed system lies in feature engineering within the SVM framework, which enhances interpretability and facilitates a better understanding of model behavior, distribution shift, and the impact of SMOTE in the feature space. This increased transparency supports the practical adoption. We also successfully identify ambiguous data samples that are prone to misclassifications by leveraging the SVM’s decision mechanism, thereby enhancing the reliability of the fault prediction system after deployment. Furthermore, this work provides a solid foundation for developing a continual learning framework for fault prediction in SIPs, aimed at iteratively updating the model with new operational data to maintain high performance in dynamic environments.

## Figures and Tables

**Figure 11:**
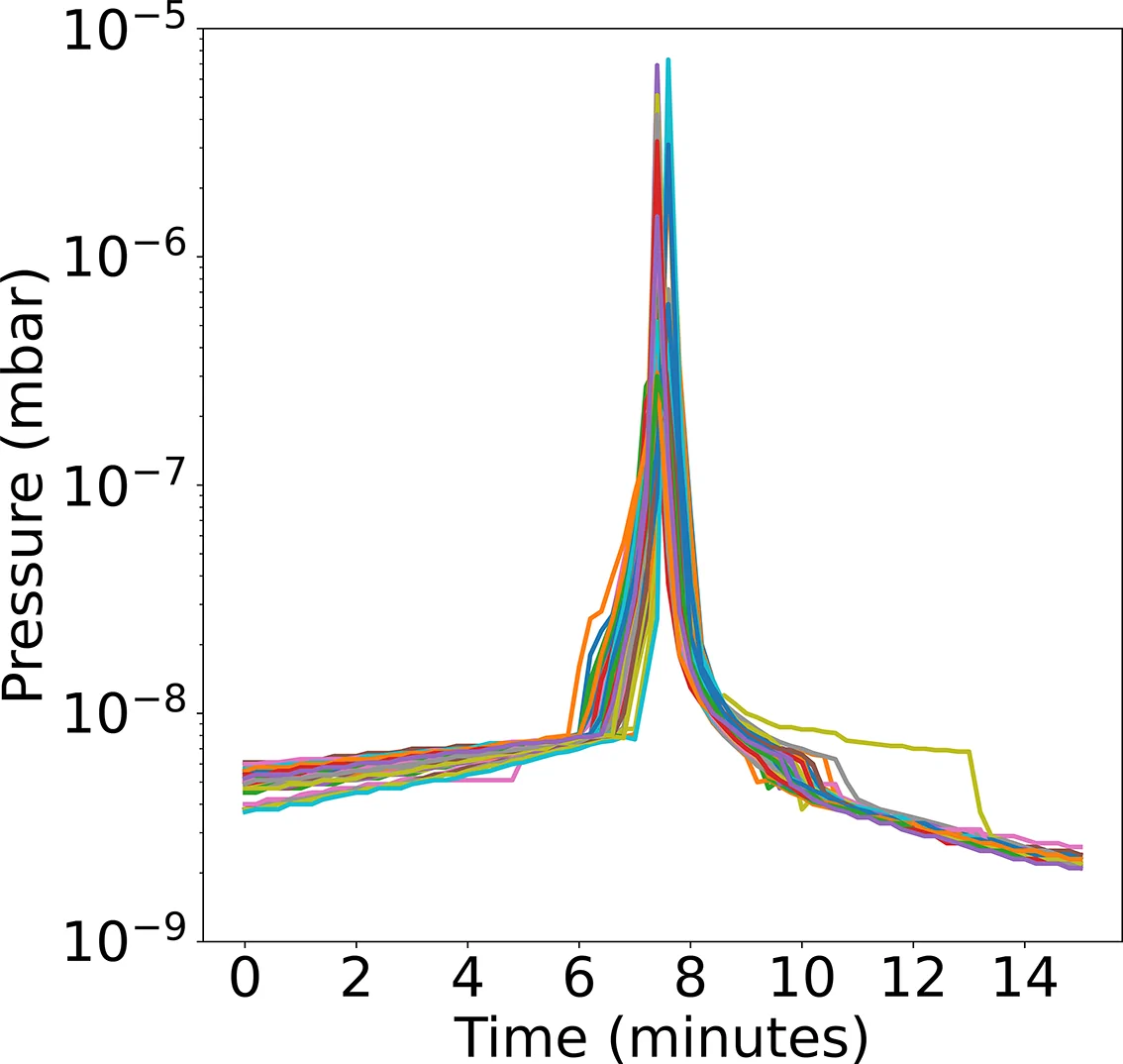
Plot of pressure measurements for all 115 occurrences of NGI.

**Figure 12:**
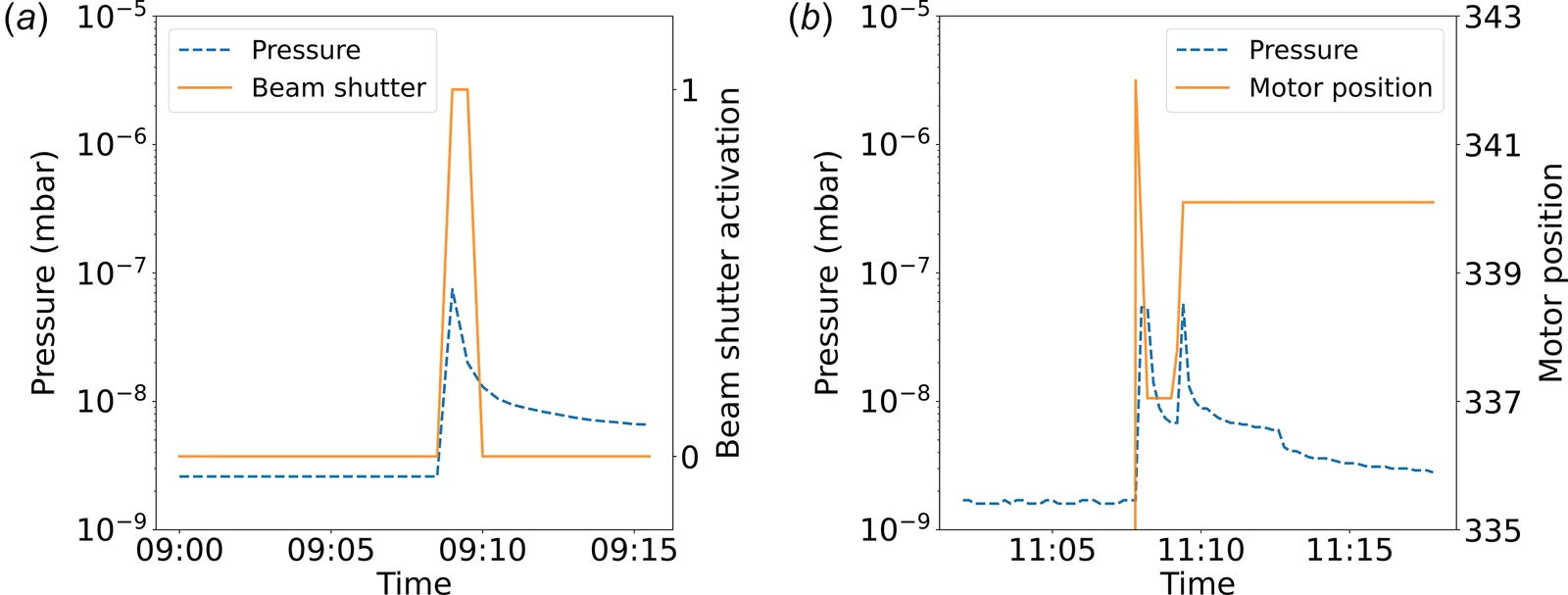
Pressure data examples from Normal category. (*a*) Peaks in pressure due to beam shutter activation. (*b*) Pressure fluctuations associated with the K-monochromator movement in the beamline.

**Figure 14:**
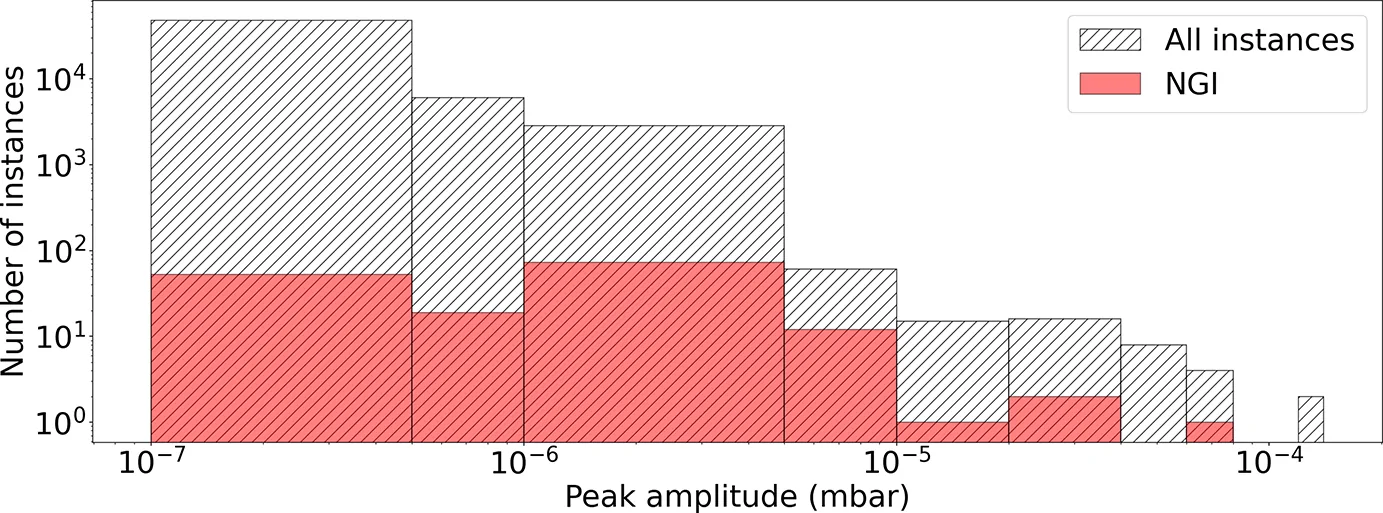
Histogram of peak pressure values across the collected 15 min pressure time-series instances with peak pressures greater than or equal to 1 × 10^−7^ mbar. Red bars represent instances associated with NGI events, whereas white bars represent all instances satisfying this threshold.

**Table 1:** Datasets overview

	Training set			Test set		
Dataset	# Normal	# NGI	Faulty pump	# Normal	# NGI	Faulty pump
DS-I	10449	80	**p1** and **p2**	4478	35	**p1** and **p2**
DS-II	10449	17	**p1**	4478	98	**p2**
DS-III	10449	98	**p2**	4478	17	**p1**
DS-IV	10449	9	**p2**	4478	106	**p1** and **p2**

**Table 2:** F1-score for the NGI class for samples that fall outside the margin of the SVM classifier; for samples within the margin, domain expert review is required, and the associated manual assessment cost is quantified as the number of samples reviewed

	Technique for imbalanced data			
	Cost-sensitive learning		SMOTE + cost-sensitive learning	
ML model	F1-score ( 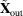 )	Cost (# of samples in 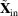 )	F1-score ( 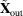 )	Cost (# of samples in 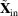 )
SVM (linear kernel)	0.70	21	0.69	22
SVM (RBF kernel)	0.71	125	0.98	34
SVM (polynomial kernel)	0.25	2	0.88	24

**Table 3:** Classification results of ML models showing precision, recall and F1-score of minority class (NGI), computed for test sets of DS-I, DS-II, DS-III and DS-IV (see Table 1 ▸)

		Technique for imbalanced data					
		Cost-sensitive learning			SMOTE + cost-sensitive learning		
Dataset	ML model	Precision	Recall	F1-score	Precision	Recall	F1-score
DS-I	SVM (linear kernel)	0.95	1.00	0.97	0.94	0.97	0.96
	SVM (RBF kernel)	0.95	1.00	0.97	0.95	1.00	0.97
	SVM (polynomial kernel)	0.97	1.00	0.99	0.97	1.00	0.99
	CNN	0.81	1.00	0.90			

DS-II	SVM (linear kernel)	1.00	0.78	0.87	1.00	0.77	0.87
	SVM (RBF kernel)	1.00	0.50	0.67	1.00	0.74	0.85
	SVM (polynomial kernel)	1.00	0.84	0.91	1.00	0.84	0.91
	CNN	0.92	1.00	0.96			

DS-III	SVM (linear kernel)	0.94	1.00	0.97	0.94	1.00	0.97
	SVM (RBF kernel)	0.94	1.00	0.97	0.94	1.00	0.97
	SVM (polynomial kernel)	1.00	0.94	0.97	1.00	0.94	0.97
	CNN	0.71	1.00	0.83			

DS-IV	SVM (linear kernel)	0.99	0.93	0.96	0.99	0.93	0.96
	SVM (RBF kernel)	1.00	0.33	0.50	0.97	1.00	0.99
	SVM (polynomial kernel)	0.98	0.93	0.96	0.99	0.95	0.97
	CNN	0.96	1.00	0.98			

**Table 4:** Post-deployment performance evaluation: classification results of ML models showing precision, recall and F1-score for minority class (NGI), computed for new data after deployment

	Technique for imbalanced data					
	Cost-sensitive learning			SMOTE + cost-sensitive learning		
ML model	Precision	Recall	F1-score	Precision	Recall	F1-score
SVM (linear kernel)	0.58	0.83	0.68	0.58	0.83	0.68
SVM (RBF kernel)	0.44	0.59	0.50	0.78	0.83	0.80
SVM (polynomial kernel)	0.67	0.17	0.28	0.77	0.89	0.83
CNN	0.77	0.50	0.61			

## Data Availability

Data and code supporting the findings of this study are available at the following URL: https://git.xfel.eu/machineLearning/vacfault. With that, the results presented in this paper can be reproduced.

## Appendix A Dataset examples

### A1. Examples from the NGI category

Fig. 11 ▸ presents a plot of pressure measurements for all 115 instances of peaks occurred due to the NGI effect. To determine an appropriate window size for pressure measurement, we manually inspected trends in pressure data and consulted with vacuum experts. In each case, a 15 min duration effectively captured the patterns associated with NGI.

### A2. Examples from the Normal category

Fig. 12 ▸ shows examples of pressure peaks from the Normal category, both resulting from deliberate operational adjustments. For instance, the peak in pressure, shown in Fig. 12 ▸(*a*), was caused by activation of the beam shutter, a component inserted into the beamline to block the X-ray beam. Its movement can generate pressure spikes due to the outgassing of beam absorber in the ultra-high-vacuum environment. Fig. 12 ▸(*b*) shows pressure fluctuations correlated with the movement of the K-monochromator (Freund *et al.*, 2019 ▸) motor. When its parts are moved into the beamline, outgassing of its material can increase the pressure level.

Identifying the root cause of pressure fluctuations such as those in Fig. 12 ▸ is challenging. Correlating pressure variations with an entire range of operational parameters requires extensive data collection and close collaboration among experts. With thousands of instruments and devices distributed along the 3 km long vacuum system at the European XFEL, isolating the exact cause of each pressure peak can be highly complex. In principle, such information could be incorporated into the ML model for fault prediction. However, given the difficulty of reliably determining all causal factors, it is extremely valuable if the classification between NGI and Normal events can be performed by analyzing the pressure data only, which is the focus of this study.

## Appendix B Feature extraction

For each data sample **x**_d_, that corresponds to a 15 min segment of pressure time series, we calculated the mean (μ_d_), standard deviation (σ_d_), skewness, kurtosis and hyperskewness described as follows,

where *x*_*j*_ is the *j*th component of a data sample **x**_*d*_,

## Appendix C Performance metrics

As described in Section 3.3, the dataset under consideration exhibits high imbalance in class distribution with significantly fewer instances representing NGI compared with Normal instances. This raises concerns regarding the adequacy of NGI representation for robust model evaluation. Consequently, we assess the performance of models using the following metrics, whose best value equals 1. The first metric, precision, measures the ratio of true positive predictions to the sum of True Positives and False Positives. It is represented by the following formula,

The second metric, recall, signifies the true positive rate, defined as

Lastly, F1-score combines precision and recall into a harmonic mean,

For all the metrics above, the ‘Positive’ class corresponds to the minority or NGI class in our study.

## Appendix D Classification results

The detailed results of the experiments described in Section 5 are reported in Tables 3 ▸ and 4 ▸.

### d1. Examples of model predictions in nearby pumps

Fig. 13 ▸ shows three examples of detected events in neighboring pumps **p11** and **p35**. As discussed in Section 5.2.3, when an NGI related pressure peak occurs in one pump, a similar response may also be observed in a neighboring pump. In such cases, the model identifies potentially relevant NGI pressure patterns, while the final assessment of the affected pump and the need for maintenance is made by vacuum experts. Accordingly, both types of responses are retained, as they may provide useful information related to NGI activity.

## Appendix E Comparison with a simple peak-detection approach

To estimate the manual inspection effort required when using a simple peak-detection approach, we examined the peak amplitudes across all available NGI instances, each representing a 15 min window of pressure time series data. The peak pressures of the NGI instances ranged from 1.6 × 10^−7^ mbar to 6.3 × 10^−5^ mbar. The distribution of peak pressures across the data instances within this range is presented in Fig. 14 ▸.

Based on the observed NGI peak-pressure range, we applied a threshold of 1 × 10^−7^ mbar to all 1126034 candidate instances obtained during data collection after deployment. This resulted in 43297 candidate instances that would require manual review when using a simple peak-detection approach. In comparison, only 53 instances required inspection using the proposed ML-based approach. The corresponding reduction in manual inspection effort is therefore

Thus, the proposed approach reduced the number of instances requiring manual inspection by more than 99% compared with this simple peak-detection approach.

## References

[bb1] Apollonio, A. (2015). *Machine protection: Availability for particle accelerators.* PhD thesis, CERN, Geneva, Switzerland.

[bb2] Branlard, J., Eichler, A., Timm, J. & Walker, N. (2022). *Proceedings of the 31st International Linear Accelerator Conference (LINAC22)*, Liverpool, UK, pp. 798–801. THPOPA26.

[bb4] Carvalho, T. P., Soares, F. A. A. M. N., Vita, R., Francisco, R. P., Basto, J. P. & Alcalá, S. G. S. (2019). *Comput. Ind. Eng.* **137**, 106024.

[bb5] Chawla, N. V., Bowyer, K. W., Hall, L. O. & Kegelmeyer, W. P. (2002). *JAIR* **16**, 321–357.

[bb6] Cortes, C., Mao, A., Mohri, M. & Zhong, Y. (2025). *Proceedings of the Forty-second International Conference on Machine Learning (ICML2025)*, Vancouver, Canada, pp. 11255–11287.

[bb7] Decking, W., Abeghyan, S., Abramian, P., Abramsky, A., Aguirre, A., Albrecht, C., Alou, P., Altarelli, M., Altmann, P., Amyan, K., Anashin, V., Apostolov, E., Appel, K., Auguste, D., Ayvazyan, V., Baark, S., Babies, F., Baboi, N., Bak, P., Balandin, V., Baldinger, R., Baranasic, B., Barbanotti, S., Belikov, O., Belokurov, V., Belova, L., Belyakov, V., Berry, S., Bertucci, M., Beutner, B., Block, A., Blöcher, M., Böckmann, T., Bohm, C., Böhnert, M., Bondar, V., Bondarchuk, E., Bonezzi, M., Borowiec, P., Bösch, C., Bösenberg, U., Bosotti, A., Böspflug, R., Bousonville, M., Boyd, E., Bozhko, Y., Brand, A., Branlard, J., Briechle, S., Brinker, F., Brinker, S., Brinkmann, R., Brockhauser, S., Brovko, O., Brück, H., Brüdgam, A., Butkowski, L., Büttner, T., Calero, J., Castro-Carballo, E., Cattalanotto, G., Charrier, J., Chen, J., Cherepenko, A., Cheskidov, V., Chiodini, M., Chong, A., Choroba, S., Chorowski, M., Churanov, D., Cichalewski, W., Clausen, M., Clement, W., Cloué, C., Cobos, J. A., Coppola, N., Cunis, S., Czuba, K., Czwalinna, M., D’Almagne, B., Dammann, J., Danared, H., de Zubiaurre Wagner, A., Delfs, A., Delfs, T., Dietrich, F., Dietrich, T., Dohlus, M., Dommach, M., Donat, A., Dong, X., Doynikov, N., Dressel, M., Duda, M., Duda, P., Eckoldt, H., Ehsan, W., Eidam, J., Eints, F., Engling, C., Englisch, U., Ermakov, A., Escherich, K., Eschke, J., Saldin, E., Faesing, M., Fallou, A., Felber, M., Fenner, M., Fernandes, B., Fernández, J. M., Feuker, S., Filippakopoulos, K., Floettmann, K., Fogel, V., Fontaine, M., Francés, A., Martin, I. F., Freund, W., Freyermuth, T., Friedland, M., Fröhlich, L., Fusetti, M., Fydrych, J., Gallas, A., García, O., Garcia-Tabares, L., Geloni, G., Gerasimova, N., Gerth, C., Geßler, P., Gharibyan, V., Gloor, M., Głowinkowski, J., Goessel, A., Gołębiewski, Z., Golubeva, N., Grabowski, W., Graeff, W., Grebentsov, A., Grecki, M., Grevsmuehl, T., Gross, M., Grosse-Wortmann, U., Grünert, J., Grunewald, S., Grzegory, P., Feng, G., Guler, H., Gusev, G., Gutierrez, J. L., Hagge, L., Hamberg, M., Hanneken, R., Harms, E., Hartl, I., Hauberg, A., Hauf, S., Hauschildt, J., Hauser, J., Havlicek, J., Hedqvist, A., Heidbrook, N., Hellberg, F., Henning, D., Hensler, O., Hermann, T., Hidvégi, A., Hierholzer, M., Hintz, H., Hoffmann, F., Hoffmann, M., Hoffmann, M., Holler, Y., Hüning, M., Ignatenko, A., Ilchen, M., Iluk, A., Iversen, J., Iversen, J., Izquierdo, M., Jachmann, L., Jardon, N., Jastrow, U., Jensch, K., Jensen, J., Jeżabek, M., Jidda, M., Jin, H., Johansson, N., Jonas, R., Kaabi, W., Kaefer, D., Kammering, R., Kapitza, H., Karabekyan, S., Karstensen, S., Kasprzak, K., Katalev, V., Keese, D., Keil, B., Kholopov, M., Killenberger, M., Kitaev, B., Klimchenko, Y., Klos, R., Knebel, L., Koch, A., Koepke, M., Köhler, S., Köhler, W., Kohlstrunk, N., Konopkova, Z., Konstantinov, A., Kook, W., Koprek, W., Körfer, M., Korth, O., Kosarev, A., Kosiński, K., Kostin, D., Kot, Y., Kotarba, A., Kozak, T., Kozak, V., Kramert, R., Krasilnikov, M., Krasnov, A., Krause, B., Kravchuk, L., Krebs, O., Kretschmer, R., Kreutzkamp, J., Kröplin, O., Krzysik, K., Kube, G., Kuehn, H., Kujala, N., Kulikov, V., Kuzminych, V., La Civita, D., Lacroix, M., Lamb, T., Lancetov, A., Larsson, M., Le Pinvidic, D., Lederer, S., Lensch, T., Lenz, D., Leuschner, A., Levenhagen, F., Li, Y., Liebing, J., Lilje, L., Limberg, T., Lipka, D., List, B., Liu, J., Liu, S., Lorbeer, B., Lorkiewicz, J., Lu, H. H., Ludwig, F., Machau, K., Maciocha, W., Madec, C., Magueur, C., Maiano, C., Maksimova, I., Malcher, K., Maltezopoulos, T., Mamoshkina, E., Manschwetus, B., Marcellini, F., Marinkovic, G., Martinez, T., Martirosyan, H., Maschmann, W., Maslov, M., Matheisen, A., Mavric, U., Meißner, J., Meissner, K., Messerschmidt, M., Meyners, N., Michalski, G., Michelato, P., Mildner, N., Moe, M., Moglia, F., Mohr, C., Mohr, S., Möller, W., Mommerz, M., Monaco, L., Montiel, C., Moretti, M., Morozov, I., Morozov, P., Mross, D., Mueller, J., Müller, C., Müller, J., Müller, K., Munilla, J., Münnich, A., Muratov, V., Napoly, O., Näser, B., Nefedov, N., Neumann, R., Neumann, R., Ngada, N., Noelle, D., Obier, F., Okunev, I., Oliver, J. A., Omet, M., Oppelt, A., Ottmar, A., Oublaid, M., Pagani, C., Paparella, R., Paramonov, V., Peitzmann, C., Penning, J., Perus, A., Peters, F., Petersen, B., Petrov, A., Petrov, I., Pfeiffer, S., Pflüger, J., Philipp, S., Pienaud, Y., Pierini, P., Pivovarov, S., Planas, M., Pławski, E., Pohl, M., Polinski, J., Popov, V., Prat, S., Prenting, J., Priebe, G., Pryschelski, H., Przygoda, K., Pyata, E., Racky, B., Rathjen, A., Ratuschni, W., Regnaud-Campderros, S., Rehlich, K., Reschke, D., Robson, C., Roever, J., Roggli, M., Rothenburg, J., Rusiński, E., Rybaniec, R., Sahling, H., Salmani, M., Samoylova, L., Sanzone, D., Saretzki, F., Sawlanski, O., Schaffran, J., Schlarb, H., Schlösser, M., Schlott, V., Schmidt, C., Schmidt-Foehre, F., Schmitz, M., Schmökel, M., Schnautz, T., Schneidmiller, E., Scholz, M., Schöneburg, B., Schultze, J., Schulz, C., Schwarz, A., Sekutowicz, J., Sellmann, D., Semenov, E., Serkez, S., Sertore, D., Shehzad, N., Shemarykin, P., Shi, L., Sienkiewicz, M., Sikora, D., Sikorski, M., Silenzi, A., Simon, C., Singer, W., Singer, X., Sinn, H., Sinram, K., Skvorodnev, N., Smirnow, P., Sommer, T., Sorokin, A., Stadler, M., Steckel, M., Steffen, B., Steinhau-Kühl, N., Stephan, F., Stodulski, M., Stolper, M., Sulimov, A., Susen, R., Świerblewski, J., Sydlo, C., Syresin, E., Sytchev, V., Szuba, J., Tesch, N., Thie, J., Thiebault, A., Tiedtke, K., Tischhauser, D., Tolkiehn, J., Tomin, S., Tonisch, F., Toral, F., Torbin, I., Trapp, A., Treyer, D., Trowitzsch, G., Trublet, T., Tschentscher, T., Ullrich, F., Vannoni, M., Varela, P., Varghese, G., Vashchenko, G., Vasic, M., Vazquez-Velez, C., Verguet, A., Vilcins-Czvitkovits, S., Villanueva, R., Visentin, B., Viti, M., Vogel, E., Volobuev, E., Wagner, R., Walker, N., Wamsat, T., Weddig, H., Weichert, G., Weise, H., Wenndorf, R., Werner, M., Wichmann, R., Wiebers, C., Wiencek, M., Wilksen, T., Will, I., Winkelmann, L., Winkowski, M., Wittenburg, K., Witzig, A., Wlk, P., Wohlenberg, T., Wojciechowski, M., Wolff-Fabris, F., Wrochna, G., Wrona, K., Yakopov, M., Yang, B., Yang, F., Yurkov, M., Zagorodnov, I., Zalden, P., Zavadtsev, A., Zavadtsev, D., Zhirnov, A., Zhukov, A., Ziemann, V., Zolotov, A., Zolotukhina, N., Zummack, F. & Zybin, D. (2020). *Nat. Photon.* **14**, 391–397.

[bb8] Dommach, M., Di Felice, M., Dickert, B., Finze, D., Eidam, J., Kohlstrunk, N., Neumann, M., Meyn, F., Petrich, M., Rio, B., Sinn, H. & Villanueva, R. (2021). *J. Synchrotron Rad.* **28**, 1229–1236. 10.1107/S1600577521005154 PMC8284404 34212888

[bb9] Freund, W., Fröhlich, L., Karabekyan, S., Koch, A., Liu, J., Nölle, D., Wilgen, J. & Grünert, J. (2019). *J. Synchrotron Rad.* **26**, 1037–1044. 10.1107/S1600577519005307 31274425

[bb10] Guler, T., Leroy, M., O’Brien, C. & Pindale, R. (2022). *Proceeedings of the 2022 Systems and Information Engineering Design Symposium (SIEDS)*, 28–29 April 2022, Charlottesville, VA, USA, pp. 397–402.

[bb11] Hauf, S., Anakkappalla, N., Taufik, J. B., Bondar, V., Costa, R., Ehsan, W., Esenov, S., Flucke, G., Garcia-Tabares, A., Giovanetti, G., *et al.* (2024). *In**Proceedings of the 19th International Conference on Accelerator and Large Experimental Physics Control Systems (ICALEPCS2023)*, 9–13 October 2023, Cape Town, South Africa, pp. 1120–1125.

[bb12] He, H. & Garcia, E. A. (2009). *IEEE Trans. Knowl. Data Eng.* **21**, 1263–1284.

[bb13] Kingma, D. P. & Ba, J. (2014). *arXiv*:1412.6980.

[bb14] Lloyd, S. (1982). *IEEE Trans. Inf. Theory* **28**, 129–137.

[bb15] Maccarrone, C., Manassero, P. & Paolini, C. (2020). *arXiv*:2006.02721.

[bb16] Nassif, A. B., Talib, M. A., Nasir, Q. & Dakalbab, F. M. (2021). *IEEE Access* **9**, 78658–78700.

[bb17] Nawaz, A., Herzog né Hoffmann, C., Graßhoff, J., Pfeiffer, S., Lichtenberg, G. & Rostalski, P. (2021). *Automatisierungstechnik* **69**, 538–549.

[bb18] Noack, M. M., Zwart, P. H., Ushizima, D. M., Fukuto, M., Yager, K. G., Elbert, K. C., Murray, C. B., Stein, A., Doerk, G. S., Tsai, E. H. R., Li, R., Freychet, G., Zhernenkov, M., Holman, H. N., Lee, S., Chen, L., Rotenberg, E., Weber, T., Goc, Y. L., Boehm, M., Steffens, P., Mutti, P. & Sethian, J. A. (2021). *Nat. Rev. Phys.* **3**, 685–697.

[bb19] O’Shea, K. & Nash, R. (2015). *arXiv*:1511.08458.

[bb20] Pagano, D., Juan, M. A., Bagnato, A., Roehm, T., Bruegge, B. & Maalej, W. (2012). *Proceedings of the 34th International Conference on Software Engineering (ICSE2012)*, pp. 1437–1438. IEEE.

[bb21] Piekarski, M., Jaworek-Korjakowska, J., Wawrzyniak, A. I. & Gorgon, M. (2020). *Measurement* **165**, 108116.

[bb22] Porcelli, T., Siviero, F., Bongiorno, G. A., Michelato, P. & Pagani, C. (2015). *Vacuum* **122**, 218–221.

[bb23] Radaideh, M. I., Pappas, C., Wezensky, M., Ramuhalli, P. & Cousineau, S. (2023). *Int. J. Prognostics Heal. Manag.* **14**, https://doi.org/10.36001/ijphm.2023.v14i1.3419.

[bb24] Rahman, M. M., Carpenter, A., Iftekharuddin, K. & Tennant, C. (2024). *Mach. Learn.: Sci. Technol.* **5**, 035078.

[bb25] Rauch, E., Linder, C. & Dallasega, P. (2020). *Comput. Ind. Eng.* **139**, 105644.

[bb26] Reščič, M., Seviour, R. & Blokland, W. (2022). *Nucl. Instrum. Methods Phys. Res. A* **1025**, 166064.

[bb27] Salcedo-Sanz, S., Rojo-Álvarez, J. L., Martínez-Ramón, M. & Camps-Valls, G. (2014). *Data Min. Knowl. Discov.* **4**, 234–267.

[bb28] Schulz, L. (1999). *Sputter-ion pumps.* Technical Report. CERN, Geneva, Switzerland.

[bb29] Sulc, A., Eichler, A. & Wilsken, T. (2023). *J. Phys. Conf. Ser.* **2420**, 012070.

[bb30] Tschentscher, T. (2023). *Eur. Phys. J. Plus* **138**, 274.

[bb31] Vapnik, V. (1995). *The Nature of Statistical Learning Theory.* Springer.

[bb32] Virtanen, P., Gommers, R., Oliphant, T. E., Haberland, M., Reddy, T., Cournapeau, D., Burovski, E., Peterson, P., Weckesser, W., Bright, J., van der Walt, S. J., Brett, M., Wilson, J., Millman, K. J., Mayorov, N., Nelson, A. R. J., Jones, E., Kern, R., Larson, E., Carey, C. J., Polat, İ., Feng, Y., Moore, E. W., VanderPlas, J., Laxalde, D., Perktold, J., Cimrman, R., Henriksen, I., Quintero, E. A., Harris, C. R., Archibald, A. M., Ribeiro, A. H., Pedregosa, F., van Mulbregt, P., Vijaykumar, A., Bardelli, A. P., Rothberg, A., Hilboll, A., Kloeckner, A., Scopatz, A., Lee, A., Rokem, A., Woods, C. N., Fulton, C., Masson, C., Häggström, C., Fitzgerald, C., Nicholson, D. A., Hagen, D. R., Pasechnik, D. V., Olivetti, E., Martin, E., Wieser, E., Silva, F., Lenders, F., Wilhelm, F., Young, G., Price, G. A., Ingold, G., Allen, G. E., Lee, G. R., Audren, H., Probst, I., Dietrich, J. P., Silterra, J., Webber, J. T., Slavič, J., Nothman, J., Buchner, J., Kulick, J., Schönberger, J. L., de Miranda Cardoso, J. V., Reimer, J., Harrington, J., Rodríguez, J. L. C., Nunez-Iglesias, J., Kuczynski, J., Tritz, K., Thoma, M., Newville, M., Kümmerer, M., Bolingbroke, M., Tartre, M., Pak, M., Smith, N. J., Nowaczyk, N., Shebanov, N., Pavlyk, O., Brodtkorb, P. A., Lee, P., McGibbon, R. T., Feldbauer, R., Lewis, S., Tygier, S., Sievert, S., Vigna, S., Peterson, S., More, S., Pudlik, T., Oshima, T., Pingel, T. J., Robitaille, T. P., Spura, T., Jones, T. R., Cera, T., Leslie, T., Zito, T., Krauss, T., Upadhyay, U., Halchenko, Y. O. & Vázquez-Baeza, Y. (2020). *Nat. Methods* **17**, 261–272.

